# Individualized Prediction of Drug Response and Rational Combination Therapy in NSCLC Using Artificial Intelligence–Enabled Studies of Acute Phosphoproteomic Changes

**DOI:** 10.1158/1535-7163.MCT-21-0442

**Published:** 2022-04-03

**Authors:** Elizabeth A. Coker, Adam Stewart, Bugra Ozer, Anna Minchom, Lisa Pickard, Ruth Ruddle, Suzanne Carreira, Sanjay Popat, Mary O'Brien, Florence Raynaud, Johann de Bono, Bissan Al-Lazikani, Udai Banerji

**Affiliations:** 1 Department of Data Science, The Institute of Cancer Research, London, United Kingdom.; 2 Wellcome Sanger Institute, Hinxton, United Kingdom.; 3 Healx Ltd., Cambridge, United Kingdom.; 4 Division of Clinical Studies, The Institute of Cancer Research, London, United Kingdom.; 5 Division of Cancer Therapeutics, The Institute of Cancer Research, London, United Kingdom.; 6 The Royal Marsden NHS Foundation Trust, London, United Kingdom.

## Abstract

We hypothesize that the study of acute protein perturbation in signal transduction by targeted anticancer drugs can predict drug sensitivity of these agents used as single agents and rational combination therapy. We assayed dynamic changes in 52 phosphoproteins caused by an acute exposure (1 hour) to clinically relevant concentrations of seven targeted anticancer drugs in 35 non–small cell lung cancer (NSCLC) cell lines and 16 samples of NSCLC cells isolated from pleural effusions. We studied drug sensitivities across 35 cell lines and synergy of combinations of all drugs in six cell lines (252 combinations). We developed orthogonal machine-learning approaches to predict drug response and rational combination therapy. Our methods predicted the most and least sensitive quartiles of drug sensitivity with an AUC of 0.79 and 0.78, respectively, whereas predictions based on mutations in three genes commonly known to predict response to the drug studied, for example, *EGFR*, *PIK3CA*, and *KRAS*, did not predict sensitivity (AUC of 0.5 across all quartiles). The machine-learning predictions of combinations that were compared with experimentally generated data showed a bias to the highest quartile of Bliss synergy scores (*P* = 0.0243). We confirmed feasibility of running such assays on 16 patient samples of freshly isolated NSCLC cells from pleural effusions. We have provided proof of concept for novel methods of using acute *ex vivo* exposure of cancer cells to targeted anticancer drugs to predict response as single agents or combinations. These approaches could complement current approaches using gene mutations/amplifications/rearrangements as biomarkers and demonstrate the utility of proteomics data to inform treatment selection in the clinic.

## Introduction

Non–small cell lung cancer (NSCLC) is the leading cause of cancer-related mortality (1) and is an example of a tumor type that benefits from molecularly targeted treatments (2). Genomic biomarkers of sensitivity to molecularly targeted drugs used to treat NSCLC include mutations or rearrangements in *EGFR* (3), *ALK* (4), *MET* (5), *ROS* (6) and *RET* (7), and *KRAS* (8). However, more than 50% of patients with NSCLC lack gene mutations or rearrangements that can be treated with licensed anticancer drugs targeting the specific genomic aberration (2). Finding new approaches for using existing novel anticancer drugs is thus an urgent unmet need.

Preclinical discovery of biomarkers of sensitivity of cancers to targeted anticancer drugs have relied heavily on concerted efforts to link drug sensitivity to mutations in large cell line panels (9). This has been transformative in enabling precision medicine paradigms to be used in the clinic, but has limitations and needs improvement (10). Interestingly, only approximately 40 drugs currently have FDA-approved or cleared companion diagnostics across all targeted drugs (11) with NSCLC as a leading example of a disease type with biomarkers of response such as *EGFR, ALK, MET, KRAS, ROS*, and *RET* mutation/rearrangements. Gene-silencing technologies such as siRNA and CRISPR are the focus in finding determinants of resistance. For example, siRNA and CRISPR screens have identified *NF1* loss or *RIC8A* as being related to EGFR inhibitor resistance (12, 13). Proteomic profiling is another approach used to discover new biomarkers of sensitivity to targeted therapy in NSCLC: This approach has revealed novel phosphorylation sites of EGFR Y1197 and other proteins such as MAPK7 and DAP1 (14); however, this has not yet resulted in change of clinical practice. Use of historical samples or patient derived model systems to profile signaling pathways to suggest sensitivity of NSCLC to drugs such as PI3K inhibitors have been published, but these have not been used to make decisions on individual patients (15, 16).

Synergistic combination therapy is critical to overcome primary and secondary drug resistance to targeted anticancer drugs (17). Large-scale, preclinical drug combination experiments across large cell line panels (including NSCLC cell lines) have been published and been helpful in understanding biology of drug resistance (18–20). Gene silencing technologies have suggested a few testable combinations of targeted therapy in NSCLC, for example, SHP2 and ALK inhibitors (21), FGFR and m-TOR inhibitors (22), or FGFR and EGFR inhibitors (23). However, the majority of such screens identify genes related to resistance that do not have drugs that can effectively target them, and thus cannot currently be tested in the clinical setting. Other approaches focusing on signal transduction have resulted in testable combinations in NSCLC, such as EGFR and BCL6 (24) inhibitors, or MEK and AKT inhibitors (25, 26). These predictions are made on observations in cell line models and not samples of tumors obtained contemporaneously from patients, and thus have not been used to predict combination therapy in individual patients. In addition, network biology-based approaches have been used to model multi-omics networks to describe synthetic lethal target interactions in lung cancer, yet this approach does not use real drug response data in building and refining models (27). Despite these wide ranging efforts only two combination of targeted agents, that is, dabrafenib in combination with trametinib (28), and erlotinib in combination with ramucirumab (29) have been licensed for the treatment with NSCLC, whereas multiple combinations of chemotherapy and immunotherapy are used as standard of care.

Experimental approaches of drug screening, gene silencing or proteomic studies to discover biomarkers of sensitivity or rational combination therapies have provided useful research insights. However, their utility for clinical decision making is hampered because they use technology for use in cell lines that either require experimental techniques like long-term cell culture and drug treatment (drug screens), cell transfections (siRNA/CRISPR) or large quantities of protein and extended analysis (mass spectroscopy). These limitations preclude use rapid testing of tumor samples from an individual patient against multiple drugs to enable decision making at any point in their treatment.

Here, we quantify dynamic signaling responses within cancer cells to predict drug sensitivity and rational combinations in NSCLC. The approach is applicable both to cancer cell lines and *ex vivo* to patient cells. The clinically relevant concentrations and the short exposure of drugs used in these experiments are key to clinical translation of these assays. We establish proof of concept that such an approach is feasible and, in the future, may result in the establishment of platforms that will inform clinical decision making and personalized treatment within 24–48 hours of a biopsy of individual tumors.

## Materials and Methods

### Cell lines and media

Thirty-five NSCLC cell lines were obtained from the ATCC or from collaborators and STR typed (details in Supplementary Table S1).

All cell lines were grown in RPMI-1640 (11835–063, Gibco) except for SK-LU-1 that was grown in DMEM (D5671, Sigma-Aldrich). In addition, all media were supplemented with 10% FBS (10270–106, Gibco), 1 mmol/L l-glutamine (25030–024, Gibco) and 1x MEM non-essential amino acid solution (M7145, Sigma-Aldrich). Cells were incubated at 37^o^C with 5% CO_2_. All cell lines used in experiments were between 4 and 28 passages. Cell lines were tested for *Mycoplasma* using MycoAlert (LT-07–218, Lonza) within 2 weeks before use.

### Drugs

Were obtained from Selleck chemicals. Drug concentrations used for our Luminex assays were based off the clinical maximum plasma concentration (*C*_max_) normalized to the protein binding effect in 20% FBS media: Details are provided in the Supplementary Methods.

### Luminex suspension bead assay

Cells were grown in 25 cm^2^ tissue culture flasks (Corning Inc.) at 20% FBS until approximately 80% confluent then dosed with one of seven drugs (plus 3 DMSO controls) for 1 hour. Lysate was stored at −80°C until required.

MILLIPLEX MAP Akt/mTOR phosphoprotein kit, MILLIPLEX MAPK/SAPK signaling kit, MILLIPLEX MAP RTK phosphoprotein kit (48–611MAG, 48–660MAG, HPRTKMAG-01K, respectively, Merck-Millipore) were combined with the following single-plex magnetic bead sets to produce three multiplex Luminex assays: phospho-NFkB, phospho-SRC, phospho-STAT3, phospho-STAT5 A/B, total HSP27 and GAPDH (46–702MAG, 46–710MAG, 46–623MAG, 46–641MAG, 46–608MAG, 46–667MAG, MerckMillipore). Bio-Plex Pro phospho-PDGFRa, phospho-PDGFRb and Akt (Thr308; 171-V50017M, 171-V50018M, 171-V50002, Bio-Rad) were combined into a triplex assay. Manufacturer's protocols were followed throughout.

### Cytotoxicity assays

Growth inhibition was assessed using 72-hour Sulforhodamine B (SRB) assay (details in Supplementary Methods).

### Isolation of cancer cells from patient effusions

Up to 1,000 mL of ascites or pleural fluid was collected by the patient and immunomagentically separated using previously published methods (30).

### Ethics and consent

All patients who had pleural effusions drained for palliative purposes. Pleural fluid was used in the study after investigators has obtained written informed consent. The tissue collection protocols were approved by the Institutional Review Boards and conducted in accordance with the Decleration of Helsinki.

### Bioinformatic/statistical analysis

To standardize the phosphoproteomic measurements, the control GAPDH measurements were normalized and median-centered, all other data normalized accordingly (see Supplementary Methods).

For predictions and feature selection, we created and assessed the performance of a suite of AI-based predictors. First, we used Random Forest recursive feature selection to define the phosphoprotein changes that most contributed to prediction, then trained and validated Random Forest classifiers and regressor functions (details of implementation in Supplementary Methods). Moreover, we in addition used elastic net predictors to predict responses to drugs. Similar models were constructed using notable clinical genomic features of NSCLC to allow comparisons of model performance using the different feature types in predicting drug response.

The environmental perturbation score (EPS) is an integrative function across the protein–protein interaction network neighbors. The protein networks were constructed using the highly curated interactome from canSAR (31). The absolute values of change were then integrated for the environment of each node, and then used to predict which drug target to select to produce a beneficial drug combination response. Details are in Supplementary Methods.

Combinations of drugs were assessed using Bliss independence analysis to study synergy. Details in Supplementary Data. The different distribution of the EPS rankings in the highest and lowest quartiles of the combinations ranked by the Bliss independent analysis was tested by a Mann–Whitney *U* test. Details in Supplementary Methods.

### Data availability

Data generated in this study are available upon request from the corresponding author.

## Results

### Prediction of sensitivity to targeted therapy using focused phosphoproteomic screen

**Figure 1.**
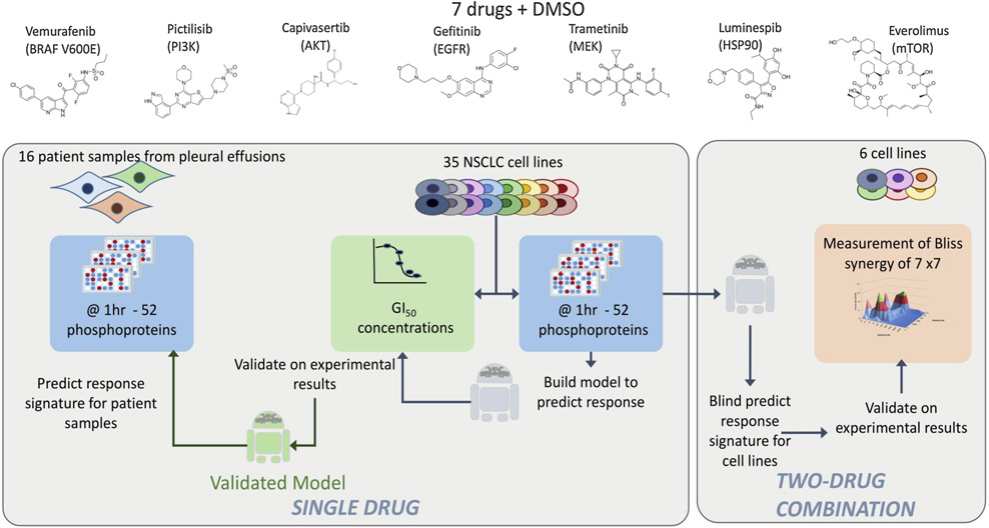
Experimental design. Single-drug evaluation: A library of 7 targeted anticancer drugs was used. First, GI_50_ concentrations were determined in a panel of 35 NSCLC cell lines with diverse genetic backgrounds (44). Second, phosphoproteomic changes of 52 selected proteins were measured after 1 hour of drug exposure of the drugs at clinically relevant concentrations adjusted for protein binding and DMSO controls were measured. The phosphoproteomic protein changes were used to train machine learning predictors of sensitivity, and validated using 100-fold cross validation with a rotating set of 15% leave out for validation and 85% for training (see Materials and Methods). The same phosphoproteomic measurements were also carried out in 16 patient samples obtained from pleural effusions producing profiles that can be fed into the predictive model to predict likely response to each drug of the individual patient samples. Two-drug combination: A novel machine learning method (environmental perturbation score) using dynamic phosphoprotein data 35 cell lines exposed to the 7 drugs was used to predict combinations. All pair wise two-drug combinations (7 individual drugs) were tested in 6 representative NSCLC cell lines and Bliss synergy was calculated for all combinations. The predicted results from the environmental perturbation score was compared with the experimentally validated results.

**Figure 2.**
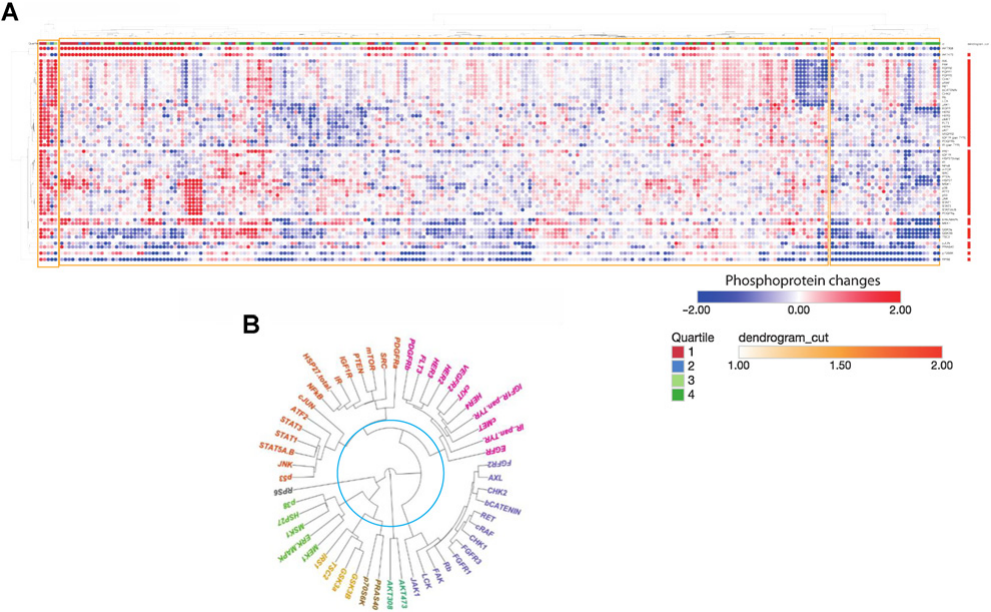
Acute dynamic phosphoproteomic perturbation. **A,** Hierarchically clustered heatmap showing 52 phosphoproteomic changes measures across all 35 cell lines exposed to all seven drugs, overlaid with quartiled drug sensitivity annotation, generated using Morpheus. Blue denotes a decreased phosphoprotein, and red denotes an increased phosphoprotein. Drug sensitivity quartiles are as illustrated and discussed in Fig. 3A. Clusters are highlighted with yellow boxes. **B,** Unrooted dendrogram representing clustering of phosphorylated proteins measured across entire dataset, showing that receptor tyrosine kinases cluster together. Colors represent distinct clusters of the dendrogram, as per slicing at the level annotated by the turquoise line.

We experimentally profiled 35 NSCLC cell lines (Supplementary Table S1) and 16 samples of immunomagnetically separated cancer cells from patients with NSCLC with pleural effusions. Cells were exposed to a single concentration (*C*_max_ adjusted for protein binding in culture medium) of 7 anticancer drugs: Gefitinib (EGFRi), trametinib (MEKi), pictilisib (PI3Ki), capivasertib (AKTi), everolimus (m-TORi), vemurafenib (BRAFi), and luminespib (HSP90i) for 1 hour to recapitulate a clinical setting and eventual translational relevance of our experiments. We chose a limited panel of drugs with well-understood mechanisms of action that had been either licensed or evaluated in clinical trials. We used a panel of 52 relevant phosphoproteins based on the known action of our drug panel and previously validated signal transduction pathways. Using highly curated protein–protein interaction data (31, 32), we constructed a protein–protein interaction network to act as a framework to map and interpret our experimental data (Supplementary Fig. S1). We chose to use an early time point and this antibody-based platform (33, 34) because it would serve as a prototype of an assay in a clinical setting with a possibility of generating results to inform treatment within 24–48 hours. The experimental design and analysis are illustrated in Fig. 1 and expanded in the on-line methods. Quantified changes in protein phosphorylation in response to 1 hour of drug incubation are shown in Fig. 2A. On average, cell lines show downregulation of 11.88 phosphoproteins (22.4% of the panel) and upregulation of 11.95 phosphoproteins (22.5% of the panel) per experimental condition, whereas patient-derived samples have on average 8.94 phosphoproteins downregulated and 13.25 phosphoproteins upregulated per experimental condition, corresponding to 16.9% and 25% of the panel, respectively. This demonstrates that in terms of number of phosphoproteins perturbed in response to drug treatment, patient-derived samples and cell lines are comparable. A dendrogram shows the clustering of the phosphoproteins based on the phosphorylation profile across the entire dataset (Fig. 2B).

We chose to compare our findings with the recently published CPPA database (35, 36) that describes similar drug perturbation using an RPPA platform on a variety of drugs and cancer cell lines. Of the seven drugs used in this study, four have also been used in the CPPA dataset (trametinib, gefitinib, vemurafenib, and pictisilib). Only one cell line was common between the CPPA database and our experiments (A549) and this cell was not exposed to any of the drugs used in our experiments. For the four common drugs in both databases, changes in 26 proteins are measured in both studies. Despite different concentrations and lengths of drug exposure, the RPPA values for these drug treatments produce similar results: Supplementary Fig. S2A shows hierarchical clustering of the data, demonstrating that the RPPA profiles do not separate by source and that many CPPA profiles are more similar to profiles generated in this study, and vice versa. Equally, Supplementary Fig. S2B shows that for the first two components of principal component analysis (PCA) analysis, the source of the data is not a major driver of variance. This indicates, in part, that the phosphoproteomic data generated in this study are broadly aligned with those currently in the public domain.

**Figure 3.**
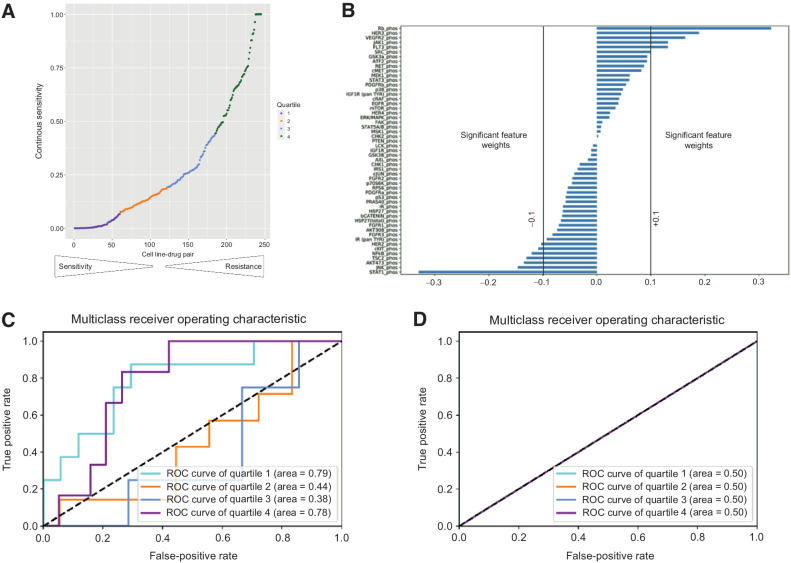
Prediction of drug sensitivity using phosphoproteomic analysis. **A,** Classification of cell line–drug single-agent sensitivities into four quartiles, with Q1 = most sensitive and Q4 = least sensitive. **B,** Feature importance of phosphoproteins based on elastic net analysis shown. Features are described previously as significant if the weight is greater than +0.1 or lesser than −0.1. **C**, Performance of predictions of sensitivity quartile based on phosphoproteomic changes using elastic net analysis. **D,** Performance of prediction of sensitivity quartile based on three clinically relevant mutations (*EGFR*, *PIK3CA*, and *KRAS*) using elastic net analysis.

We then trained a suite of orthogonal machine learning algorithms with appropriate training and validation sets (random forest regressors, classifiers and elastic net, see Supplementary Methods) to define the key phosphoprotein changes that predict drug sensitivity in individual cell lines. For comparison, we applied the same algorithms to test the power of known genomic features to predict drug sensitivity. We divided the response data into four quartiles where the first quartile and fourth quartile contain the least and most drug sensitive outcomes, respectively (Fig. 3A). Feature importance of phosphoproteins used in the elastic net analysis was described previously as significant if the absolute weight is greater than 0.1 (Fig. 3B). We find that dynamic phosphoproteomic changes can strongly predict high and low drug response (Supplementary Fig. S3) with an AUC of 0.78–0.79 for Q1 and Q4 (Fig. 3C; Supplementary Fig. S3A, S3C, and S3E). In comparison, genomic features such as mutations in *EGFR*, *KRAS*, and *PIK3C*A failed to predict sensitivity in the same samples (Fig. 3D; Supplementary Fig. S3B, S3D, and S3F). This demonstrates that dynamic proteomic profiles enable more accurate single-agent drug response prediction than the mutational statuses of *EGFR, KRAS*, and *PIK3CA*—the three genomic markers currently used in the clinic to predict drug response.

In addition, we calculated the predictive performance of each of the three mutated genes when targeted with drugs against their specific protein. Despite *EGFR* (3) and *PIK3CA* (37) mutations being used in the clinic to select patients most likely to respond to EGFR and PI3K inhibitors, we identified that *EGFR-*mutated cell lines did not show an enrichment for sensitivities to the EGFR inhibitor gefitinib in Quartiles 1 and 2 relative to the *EGFR* wild-type cell lines (χ^2^ test with Yates correction, *P* = 0.67) (Supplementary Table S2). Equally, *PIK3CA*-mutated cell lines did not show an enrichment for sensitivities to the PI3K inhibitor pictisilib in Quartiles 1 and 2 (*χ*^2^ test with Yates correction, *P* = 0.23). Although this may be due to the relatively small sample sizes of numbers of cell lines, these results highlight the limitations of using genotype alone to predict sensitivity to targeted drugs, even those that target a protein that can drive a cancer cell. These experiments were performed before KRAS G12C inhibitors becoming available; however, proteomic analysis outperformed *KRAS* mutations to predict sensitivity/resistance to all drugs studied. Thus, the predictive power of phosphoproteomic changes in the models studied shows that they could be used to augment current predictive biomarker paradigms based on genotype.

### Prediction of synergistic and antagonistic combinations using focused phosphoproteomic screening results

**Figure 4.**
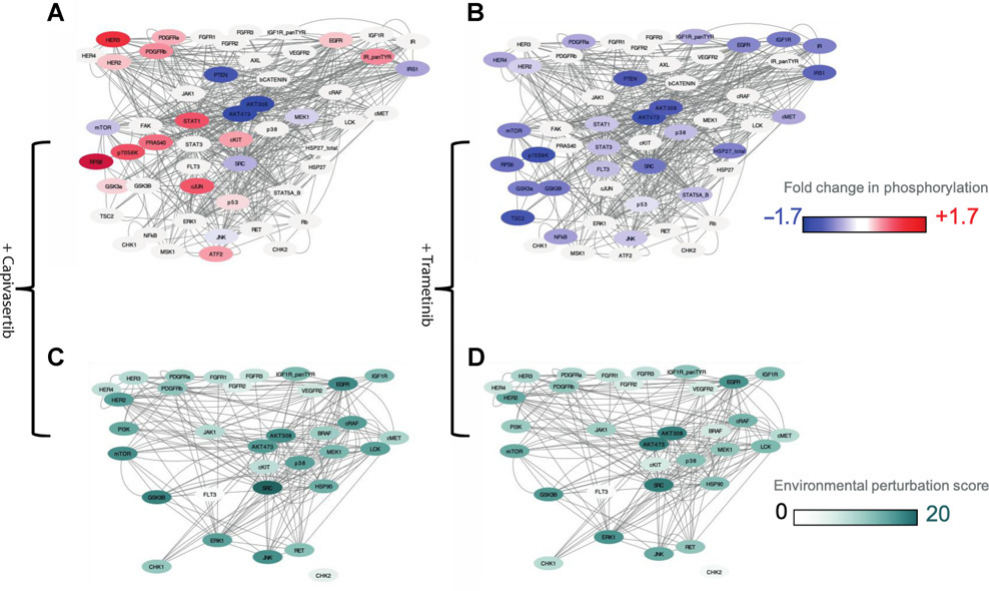
Dynamic changes in phosphoproteins and EPS. Exemplar of results in a cell line HCC827. **A** and **B,** Network diagrams showing phosphoproteomic changes and drug targets with color gradient blue (−1.7) and red (+1.7). Nodes that are drug targets but where phosphorylation has not been measured are denoted in gray, that is, HSP90, PI3K, and BRAF. **A,** Phosphoproteomic changes related to exposure to the AKT inhibitor capivasertib. **B,** Phosphoproteomic changes related to exposure to the MEK inhibitor trametinib. **C** and **D,** EPS calculated for nodes that are tractable on CanSAR. **C,** EPS scores upon exposure to the AKT inhibition capivasertib. **D,** EPS scores upon exposure the MEK inhibitor trametinib.

We applied our method of calculating dynamic EPS of each individual phosphoprotein when exposed a drug to predict synergistic combination (see Supplementary Methods for details). The list of EPS values for each node per cell line per drug is presented in the Supplementary Table S3 and an example of proteomic changes caused by capivasertib and trametinib in the HCC827 cell line and the associated EPS score are shown in (Fig. 4A–D), respectively. Note that using this measure, a node can be a signaling junction, even if its own perturbation is low.

**Figure 5.**
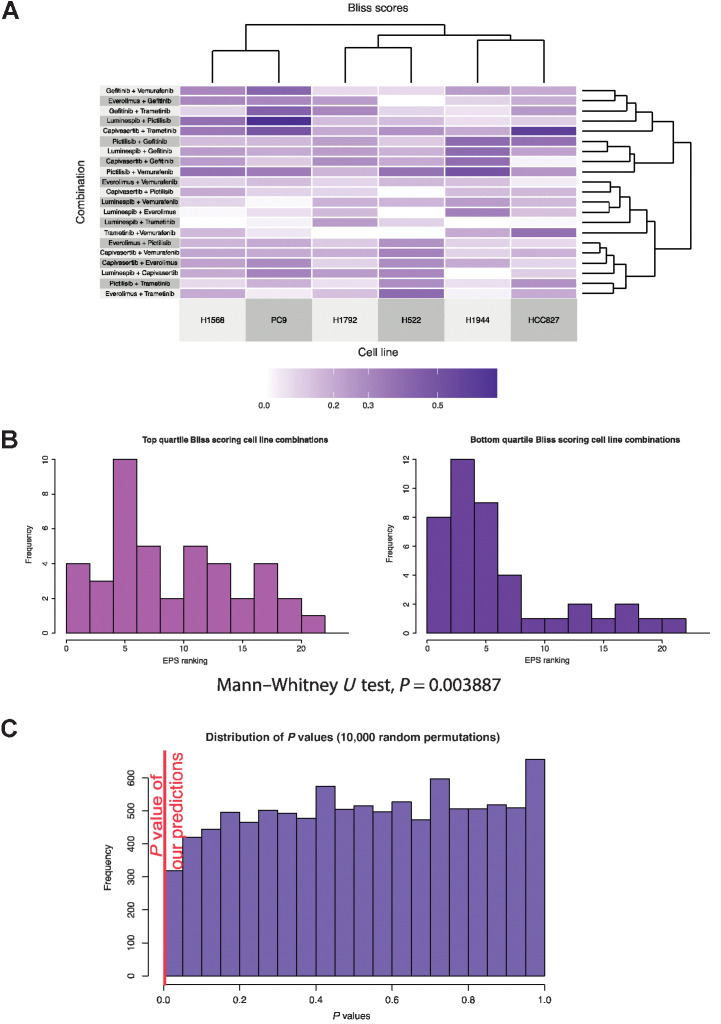
Experimental and predicted combinations. **A,** Clustered heatmap of Bliss synergy scores was experimentally measured for six cell lines treated with 21 two-drug combinations. **B,** Histogram representing the EPS rankings of nodes of targets of drugs in the top 25% highest Bliss synergy scores, that is, “most synergistic” (left), or the EPS rankings of nodes of targets of drugs in the 25% lowest Bliss synergy scores, that is, “least synergistic” (right). There is a significant bias toward higher EPS rankings for the most synergistic drug targets, with a significant Mann–Whitney *U* test *P* value of 0.0038875, indicating a biased distribution of rankings. **C,** Simulation of the Mann–Whitney *U* test *P* values obtained from 10,000-fold random permutations of EPS ranking, demonstrating the robustness of this *P* value.

To test and validate the power of the EPS in predicting synergistic combinations, we conducted blind unbiased pairwise combination screening *in vitro* of the 7 drugs in 6 cell lines (2 *EGFR* mutated, 2 *KRAS* mutated, and 2 wt for *EGFR* and *KRAS*), resulting in 252 experimentally derived Bliss independence scores. The Bliss independence scores of all the combinations in the 6 cell lines are represented in Fig. 5A, Supplementary Table S4. We show that of the 128 cell line-combination pairs with a Bliss score >0.1 (i.e., synergy), EPS correctly identified the combination to be in the top 5 ranked combinations in 73 (57%) cases and the top 10 ranked combinations in 106 (83%) cases. EPS correctly identified previously reported synergistic combinations of MEK or EGFR inhibitors with PI3K pathway inhibitors (25, 26, 38)—examples of true positive synergistic combinations. For example, EPS identified combinations of trametinib and capivasertib in HCC827 cells (Bliss 0.6, EPS ranking for AKT_308 of 3, AKT_473 of 2) and gefitinib and everolimus in PC9 cells (Bliss 0.3, EPS ranking for mTOR of 2). Moreover, EPS was able to correctly predict previously unreported combinations such as vemurafenib and capivasertib in H522 cells (Bliss 0.32, EPS ranking for AKT_308 of 4, AKT473 of 2), Supplementary Table S4.

We find that although EPS is a strong predictor of clear synergy or clear lack of synergy, it was unable to distinguish marginal signals. Thus, when counting all data, we do not observe clear correlation between the Bliss independence score and the EPS (*R*^2^ = 0.0132; Supplementary Fig. S4). However, we observed enrichment of correct predictions in the highest and lowest Bliss data quartiles: Predictions for these quartiles showed significantly skewed distributions (Mann–Whitney *U* test *P* value of 0.003887). To test the statistical significance of this enrichment, we compared the concordance of our EPS ranking with synergy based on the experimental input data versus 10,000 equivalent rankings based on randomly simulated data (see Supplementary Methods). We found a clear difference between EPS concordance with the experimental data of *P* values of <0.1 with that of the random rankings (Fig. 5A–C). This is remarkable as we used a 52 phosphoprotein panel and only generated experimental data studying growth inhibition of combinations using 7 drugs. Thus, the EPS method so far is unable to predict marginal synergistic signals, but it is very successful at predicting clear events such as clear synergy or clear lack of synergy.

### The route to clinical translation

**Figure 6.**
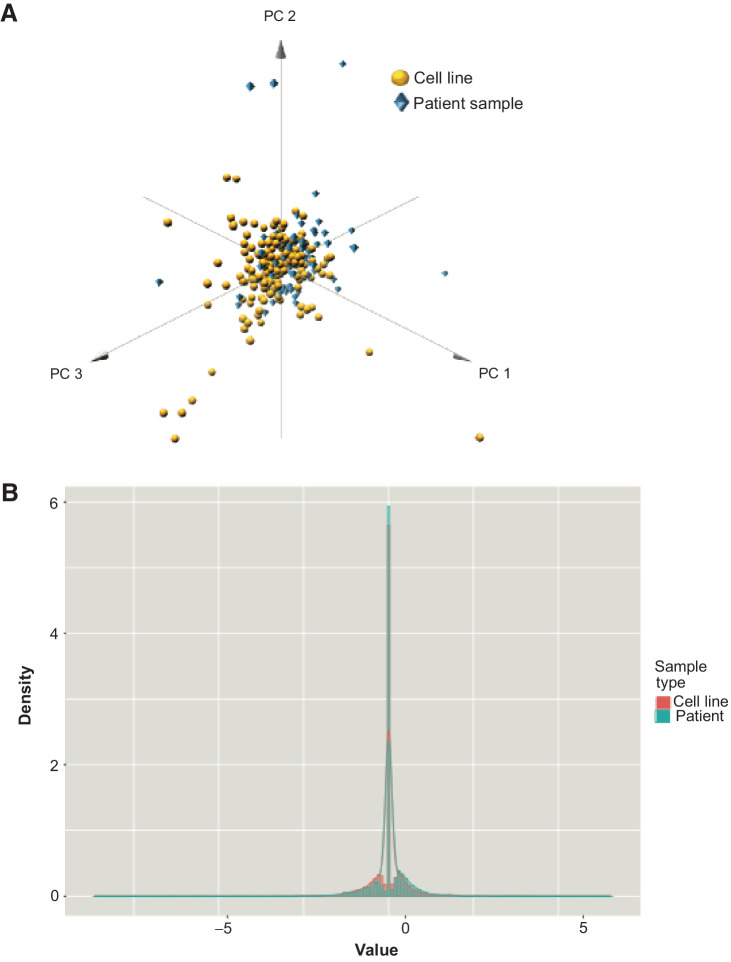
Comparison phosphoprotein changes in patient samples and cell lines. **A,** 3D plot showing that for the first three principal components of the phosphoproteomic data, patient samples (blue diamonds) show comparable distribution with cell line data (yellow circles), indicating that changes in phosphorylation in cell line panels could potentially reflect changes within clinical samples. **B,** Probability density functions of cell line and patient data, showing a strong overlap in distribution and peak values between the two sample types, despite a Welch Two Sample *t* test, indicating that the two groups have different means (*P* = 0.006804). Here, *x*-axis plots the value of dynamic phosphoprotein changes, and the *y*-axis (density) is proportional to frequency.

In keeping with our desire to translate our proof-of-concept findings to a clinically relevant platform, in addition to exposing established NSCLC cell lines clinical relevant concentrations (*C*_max_ adjusted for protein binding) for 1 hour, we exposed immunomagnetically separated cancer cells isolate from fresh pleural effusion aspirates to the 7 drugs under identical conditions. The phosphoprotein analysis was conducted and PCA of phosphoprotein changes due to 7 drugs in established NSCLC cell lines (*n* = 35) and samples from patients (*n* = 16) were broadly similar (Fig. 6A); similar results were found when plotting the probability density functions of the two sample types, despite a statistically significant difference in their distributions (Fig. 6B). It is important to note that the collection of the sample from the patient, *ex vivo* treatment for 1 hour, cell lysis, protein quantification, quantification of phosphoproteins on the antibody-based proteomic platform and machine learning analysis could technically be carried out within a 48-hour window, thus demonstrating the feasibility of this technique for use in the clinic to deliver rapid and accurate predictions of patient response, and thus inform drug selection. Significant further validation will be required before use in patients.

## Discussion

To our knowledge, we have showed for the first time that simultaneously quantifying multiple phosphoproteins responses to clinically relevant concentrations of targeted anticancer drugs for a short period of time (1 hour) can be used to predict drug sensitivity: These data were able to outperformed known genetic biomarkers as predictors of sensitivity in the cell line panels and drugs studied. The tailoring of experiments to use clinically relevant concentrations adjusted to protein binding and an acute 1 hour exposure to be used clinically on biopsy specimens in the future, make our proteomic dataset and analysis different from other important recently published work on effects of drugs on proteomic perturbation (36). However, these previously published resources are helpful to benchmark some of the changes seen in our analysis (35, 36). Although our study acts as a proof of concept, the length of time used for drug incubation could be further optimized to identify the optimal time point at which to obtain the highest predictive power of proteomic responses.

Multiple factors contribute to the need for not relying solely on genetic biomarkers such as tissue context specificity. For example, G12C KRAS inhibitors cause clinical benefit in *KRAS* G12C-driven NSCLC but not colorectal cancer and this is related to feedback loops involving EGFR signaling (39). Furthermore, we have previously shown context-specific signaling differences in signaling between NSCLC, colorectal cancer, and PDAC cell lines (34). Other factors could include transcriptional silencing of genetic aberrations (40). Finally, the challenge posed by spatial and tumor temporal heterogeneity cannot be underestimated (41).

We have also for the first time described the use of EPS in predicting synergistic combinations. We validated the model by running all possible combinations of the 7 drugs described previously in the article in six cell lines. The proteomics-based EPS model predicted synergy significantly better than over 10,000 random permutations of EPS rankings. Interestingly, some of the combinations suggested by our methodology such as the synergy of the combination of MEK and PI3K pathway inhibitors have previously been reported following specific hypothesis testing experiments (25, 26, 42), which partially confirms our findings with true positives. However, the EPS model is particularly exciting as it can discover novel combinations in an unbiased way. There have been no unbiased, systematic drug combination therapy screens reported in NSCLC to date; however, NSCLC cell lines have been included in large drug screens (18–20). Outside NSCLC, multiple approaches using gene silencing techniques such as siRNA/CRISPR have been attempted and are out of the scope of this article, but such experimental systems would need long-term cultures of patient-derived tissue to make predictions of drug response for individual patients in the clinic. In contrast, our approach uses acute incubation of patient-derived cells to make accurate and informative predictions of drug response.

In this article, a set of unbiased combination experiments, done to validate the EPS have statistically shown high concordance in the highest and lowest quartiles predictions of synergy. Predictions of top and bottom quartiles of responses represent a stepping stone from binary classifications of sensitive/insensitive and toward an ultimate goal of predictions of precise, continuous synergy. In addition, prediction of ranked sensitivities as opposed to absolute values may be of benefit when considering the well-known challenges of translating *in vitro* cell line observations into *in vivo* studies or patients (43). Although we have established early proof of concept, iterative improvements, that is, incorporating the use of larger proteomic datasets, new drugs, and newer understanding signal transduction pathways will further improve this approach.

There are biological complexities such as the role of stroma or the immune system that cannot be captured in the model system described previously in this article. However, we do believe that the current approach is a functional assay that can be delivered in the clinic, which intellectually lies in between genomics (finding mutations/amplifications/deletions or siRNA/CRISPR experiments) and truly phenotypic assays (cell culture/organoid and patient derived xenografts), with the added advantage of being able to near contemporaneously predict sensitivity and synergistic combination therapy. The EPS algorithm, based on acute phosphoproteomic changes, has been validated in *in vitro* experimental models. Although *in vivo* testing is desirable, to meaningfully impact the model (7 drugs across 35 cell line models and 252, 2 drug combinations), xenograft experiments need be done at a scale that is out the scope for academic groups. Showing the results of 1–2 xenograft models to show proof of concept, although conventional, we felt would be against the spirit of unbiased testing and thus we have not conducted these experiments for this article. Such experiments will have to be considered before using the assay in the clinical setting.

To conclude, we have demonstrated for the first time that the use of a focused phosphoproteomic assay and machine learning approaches that has used dynamic phosphorylation in signal transduction to predict sensitivity to drugs and prioritize rational combinations tested on cancer cell lines and patient samples in NSCLC. This is a powerful approach that is orthogonal to genomic markers, is adaptive and individualized, with a clinically meaningful turnaround time. This feasibility study provides proof of concept; however, considerable technical validation is needed before use in patients. If developed further, that this methodology can potentially improve the outcomes of patients with cancer treated with targeted anticancer drugs as a single agent or as combination therapy.

## Supplementary Material

Supplementary Figure

Supplementary Figure

Supplementary Figure

Supplementary Figure

Supplementary Table

Supplementary Table

Supplementary Table

Supplementary Table

Supplementary Table

## References

[1] Bray F , Ferlay J, Soerjomataram I, Siegel RL, Torre LA, Jemal A. Global cancer statistics 2018: GLOBOCAN estimates of incidence and mortality worldwide for 36 cancers in 185 countries. CA Cancer J Clin 2018;68:394–424. 30207593 10.3322/caac.21492

[2] Arbour KC , Riely GJ. Systemic therapy for locally advanced and metastatic non–small cell lung cancer: a review. JAMA 2019;322:764–74. 31454018 10.1001/jama.2019.11058

[3] Ramalingam SS , Vansteenkiste J, Planchard D, Cho BC, Gray JE, Ohe Y, . Overall survival with osimertinib in untreated, EGFR-mutated advanced NSCLC. N Engl J Med 2020;382:41–50. 31751012 10.1056/NEJMoa1913662

[4] Camidge DR , Kim HR, Ahn MJ, Yang JC, Han JY, Lee JS, . Brigatinib versus crizotinib in ALK-positive non–small cell lung cancer. N Engl J Med 2018;379:2027–39. 30280657 10.1056/NEJMoa1810171

[5] Wolf J , Seto T, Han J, Reguart N, Garon E, Groen H, . Capmatinib (INC280) in METΔex14-mutated advanced non–small cell lung cancer (NSCLC): efficacy data from the phase II GEOMETRY mono-1 study. J Clin Oncol 2019;37:9004.

[6] Wu YL , Yang JC, Kim DW, Lu S, Zhou J, Seto T, . Phase II study of crizotinib in East Asian patients with ROS1-positive advanced non–small cell lung cancer. J Clin Oncol 2018;36:1405–11. 29596029 10.1200/JCO.2017.75.5587

[7] FDA approves selpercatinib; pralsetinib may soon follow. Cancer Discov 2020;10:OF1. 10.1158/2159-8290.CD-NB2020-052 32493697

[8] Canon J , Rex K, Saiki AY, Mohr C, Cooke K, Bagal D, . The clinical KRAS(G12C) inhibitor AMG 510 drives antitumour immunity. Nature 2019;575:217–23. 31666701 10.1038/s41586-019-1694-1

[9] Garnett MJ , Edelman EJ, Heidorn SJ, Greenman CD, Dastur A, Lau KW, . Systematic identification of genomic markers of drug sensitivity in cancer cells. Nature 2012;483:570–5. 22460902 10.1038/nature11005 PMC3349233

[10] Tannock IF , Hickman JA. Limits to personalized cancer medicine. N Engl J Med 2016;375:1289–94. 27682039 10.1056/NEJMsb1607705

[11] US Food & Drug Administration. List of cleared or approved companion diagnostic devices (in vitro and imaging tools). Available from: https://www.fda.gov/medical-devices/vitro-diagnostics/list-cleared-or-approved-companion-diagnostic-devices-vitro-and-imaging-tools.

[12] Zeng H , Castillo-Cabrera J, Manser M, Lu B, Yang Z, Strande V, . Genome-wide CRISPR screening reveals genetic modifiers of mutant EGFR dependence in human NSCLC. Elife 2019;8:e50223. 31741433 10.7554/eLife.50223 PMC6927754

[13] de Bruin EC , Cowell C, Warne PH, Jiang M, Saunders RE, Melnick MA, . Reduced NF1 expression confers resistance to EGFR inhibition in lung cancer. Cancer Discov 2014;4:606–19. 24535670 10.1158/2159-8290.CD-13-0741 PMC4011693

[14] Zhang X , Maity T, Kashyap MK, Bansal M, Venugopalan A, Singh S, . Quantitative tyrosine phosphoproteomics of epidermal growth factor receptor (EGFR) tyrosine kinase inhibitor-treated lung adenocarcinoma cells reveals potential novel biomarkers of therapeutic response. Mol Cell Proteomics 2017;16:891–910. 28331001 10.1074/mcp.M117.067439 PMC5417828

[15] Spoerke JM , O'Brien C, Huw L, Koeppen H, Fridlyand J, Brachmann RK, . Phosphoinositide 3-kinase (PI3K) pathway alterations are associated with histologic subtypes and are predictive of sensitivity to PI3K inhibitors in lung cancer preclinical models. Clin Cancer Res 2012;18:6771–83. 23136191 10.1158/1078-0432.CCR-12-2347

[16] Shi R , Li M, Raghavan V, Tam S, Cabanero M, Pham NA, . Targeting the CDK4/6-Rb pathway enhances response to PI3K inhibition in PIK3CA-mutant lung squamous cell carcinoma. Clin Cancer Res 2018;24:5990–6000. 30093452 10.1158/1078-0432.CCR-18-0717

[17] Al-Lazikani B , Banerji U, Workman P. Combinatorial drug therapy for cancer in the post-genomic era. Nat Biotechnol 2012;30:679–92. 22781697 10.1038/nbt.2284

[18] O'Neil J , Benita Y, Feldman I, Chenard M, Roberts B, Liu Y, . An unbiased oncology compound screen to identify novel combination strategies. Mol Cancer Ther 2016;15:1155–62. 26983881 10.1158/1535-7163.MCT-15-0843

[19] Holbeck SL , Camalier R, Crowell JA, Govindharajulu JP, Hollingshead M, Anderson LW, . The national cancer institute ALMANAC: a comprehensive screening resource for the detection of anticancer drug pairs with enhanced therapeutic activity. Cancer Res 2017;77:3564–76. 28446463 10.1158/0008-5472.CAN-17-0489 PMC5499996

[20] Menden MP , Casale FP, Stephan J, Bignell GR, Iorio F, McDermott U, . The germline genetic component of drug sensitivity in cancer cell lines. Nat Commun 2018;9:3385. 30139972 10.1038/s41467-018-05811-3 PMC6107640

[21] Dardaei L , Wang HQ, Singh M, Fordjour P, Shaw KX, Yoda S, . SHP2 inhibition restores sensitivity in ALK-rearranged non–small cell lung cancer resistant to ALK inhibitors. Nat Med 2018;24:512–7. 29505033 10.1038/nm.4497 PMC6343825

[22] Singleton KR , Hinz TK, Kleczko EK, Marek LA, Kwak J, Harp T, . Kinome RNAi screens reveal synergistic targeting of MTOR and FGFR1 pathways for treatment of lung cancer and HNSCC. Cancer Res 2015;75:4398–406. 26359452 10.1158/0008-5472.CAN-15-0509 PMC4609283

[23] Raoof S , Mulford IJ, Frisco-Cabanos H, Nangia V, Timonina D, Labrot E, . Targeting FGFR overcomes EMT-mediated resistance in EGFR mutant non–small cell lung cancer. Oncogene 2019;38:6399–413. 31324888 10.1038/s41388-019-0887-2 PMC6742540

[24] Zhou Tran Y , Minozada R, Cao X, Johansson HJ, Branca RM, Seashore-Ludlow B, . Immediate adaptation analysis implicates BCL6 as an EGFR-TKI combination therapy target in NSCLC. Mol Cell Proteomics 2020;19:928–43. 32234966 10.1074/mcp.RA120.002036 PMC7261823

[25] Tolcher AW , Khan K, Ong M, Banerji U, Papadimitrakopoulou V, Gandara DR, . Antitumor activity in RAS-driven tumors by blocking AKT and MEK. Clin Cancer Res 2015;21:739–48. 25516890 10.1158/1078-0432.CCR-14-1901 PMC4335074

[26] Stewart A , Thavasu P, de Bono JS, Banerji U. Titration of signalling output: insights into clinical combinations of MEK and AKT inhibitors. Ann Oncol 2015;26:1504–10. 25908604 10.1093/annonc/mdv188 PMC4478974

[27] Broyde J, Simpson D, Murray D, Giorgi F, Lachmann A, Jackson P, . Systematic elucidation and validation of OncoProtein-centric molecular interaction maps. bioRxiv 2018. doi: 10.1101/289538.

[28] Planchard D , Smit EF, Groen HJM, Mazieres J, Besse B, Helland A, . Dabrafenib plus trametinib in patients with previously untreated BRAF(V600E)-mutant metastatic non–small cell lung cancer: an open-label, phase 2 trial. Lancet Oncol 2017;18:1307–16. 28919011 10.1016/S1470-2045(17)30679-4

[29] Nakagawa K , Garon EB, Seto T, Nishio M, Ponce Aix S, Paz-Ares L, . Ramucirumab plus erlotinib in patients with untreated, EGFR-mutated, advanced non–small cell lung cancer (RELAY): a randomised, double-blind, placebo-controlled, phase 3 trial. Lancet Oncol 2019;20:1655–69. 31591063 10.1016/S1470-2045(19)30634-5

[30] Carden CP , Stewart A, Thavasu P, Kipps E, Pope L, Crespo M, . The association of PI3 kinase signaling and chemoresistance in advanced ovarian cancer. Mol Cancer Ther 2012;11:1609–17. 22556379 10.1158/1535-7163.MCT-11-0996 PMC4630857

[31] Coker EA , Mitsopoulos C, Tym JE, Komianou A, Kannas C, Di Micco P, . canSAR: update to the cancer translational research and drug discovery knowledgebase. Nucleic Acids Res 2019;47:D917–D22. 30496479 10.1093/nar/gky1129 PMC6323893

[32] Mitsopoulos C , Schierz AC, Workman P, Al-Lazikani B. Distinctive behaviors of druggable proteins in cellular networks. PLoS Comput Biol 2015;11:e1004597. 26699810 10.1371/journal.pcbi.1004597 PMC4689399

[33] Stewart A , Banerji U. Utilizing the luminex magnetic bead-based suspension array for rapid multiplexed phosphoprotein quantification. Methods Mol Biol 2017;1636:119–31. 28730477 10.1007/978-1-4939-7154-1_9

[34] Stewart A , Coker EA, Polsterl S, Georgiou A, Minchom AR, Carreira S, . Differences in signaling patterns on PI3K inhibition reveal context specificity in KRAS-mutant cancers. Mol Cancer Ther 2019;18:1396–404. 31262731 10.1158/1535-7163.MCT-18-0727 PMC6679718

[35] Cancer Perturbed Proteomics Atlas. Available from: https://tcpaportal.org/cppa/#/.

[36] Zhao W , Li J, Chen MM, Luo Y, Ju Z, Nesser NK, . Large-scale characterization of drug responses of clinically relevant proteins in cancer cell lines. Cancer Cell 2020;38:829–43. 33157050 10.1016/j.ccell.2020.10.008 PMC7738392

[37] Andre F , Ciruelos E, Rubovszky G, Campone M, Loibl S, Rugo HS, . Alpelisib for PIK3CA-mutated, hormone receptor-positive advanced breast cancer. N Engl J Med 2019;380:1929–40. 31091374 10.1056/NEJMoa1813904

[38] Puglisi M , Thavasu P, Stewart A, de Bono JS, O'Brien ME, Popat S, . AKT inhibition synergistically enhances growth-inhibitory effects of gefitinib and increases apoptosis in non–small cell lung cancer cell lines. Lung Cancer 2014;85:141–6. 24957682 10.1016/j.lungcan.2014.05.008

[39] Amodio V , Yaeger R, Arcella P, Cancelliere C, Lamba S, Lorenzato A, . EGFR blockade reverts resistance to KRAS(G12C) inhibition in colorectal cancer. Cancer Discov 2020;10:1129–39. 32430388 10.1158/2159-8290.CD-20-0187 PMC7416460

[40] Adashek JJ , Kato S, Parulkar R, Szeto CW, Sanborn JZ, Vaske CJ, . Transcriptomic silencing as a potential mechanism of treatment resistance. JCI Insight 2020;5:e134824. 32493840 10.1172/jci.insight.134824 PMC7308055

[41] Siravegna G , Mussolin B, Venesio T, Marsoni S, Seoane J, Dive C, . How liquid biopsies can change clinical practice in oncology. Ann Oncol 2019;30:1580–90. 31373349 10.1093/annonc/mdz227

[42] Holt SV , Logie A, Davies BR, Alferez D, Runswick S, Fenton S, . Enhanced apoptosis and tumor growth suppression elicited by combination of MEK (selumetinib) and mTOR kinase inhibitors (AZD8055). Cancer Res 2012;72:1804–13. 22271687 10.1158/0008-5472.CAN-11-1780

[43] Wilding JL , Bodmer WF. Cancer cell lines for drug discovery and development. Cancer Res 2014;74:2377–84. 24717177 10.1158/0008-5472.CAN-13-2971

[44] Bailey MH , Tokheim C, Porta-Pardo E, Sengupta S, Bertrand D, Weerasinghe A, . Comprehensive characterization of cancer driver genes and mutations. Cell 2018;173:371–85. 29625053 10.1016/j.cell.2018.02.060 PMC6029450

