# Supplementary material for: Individualized Prediction of Drug Response and Rational Combination Therapy in NSCLC Using Artificial Intelligence–Enabled Studies of Acute Phosphoproteomic Changes
Source: Mol Cancer Ther. 2022 Apr 3;21(6):1020–9. doi: 10.1158/1535-7163.MCT-21-0442 (PMC9381105; doi:10.1158/1535-7163.MCT-21-0442)
Supplement: Supplementary Figure [file mct-21-0442_supplementary_figure_1_suppsf1.pptx]

## Slide 1
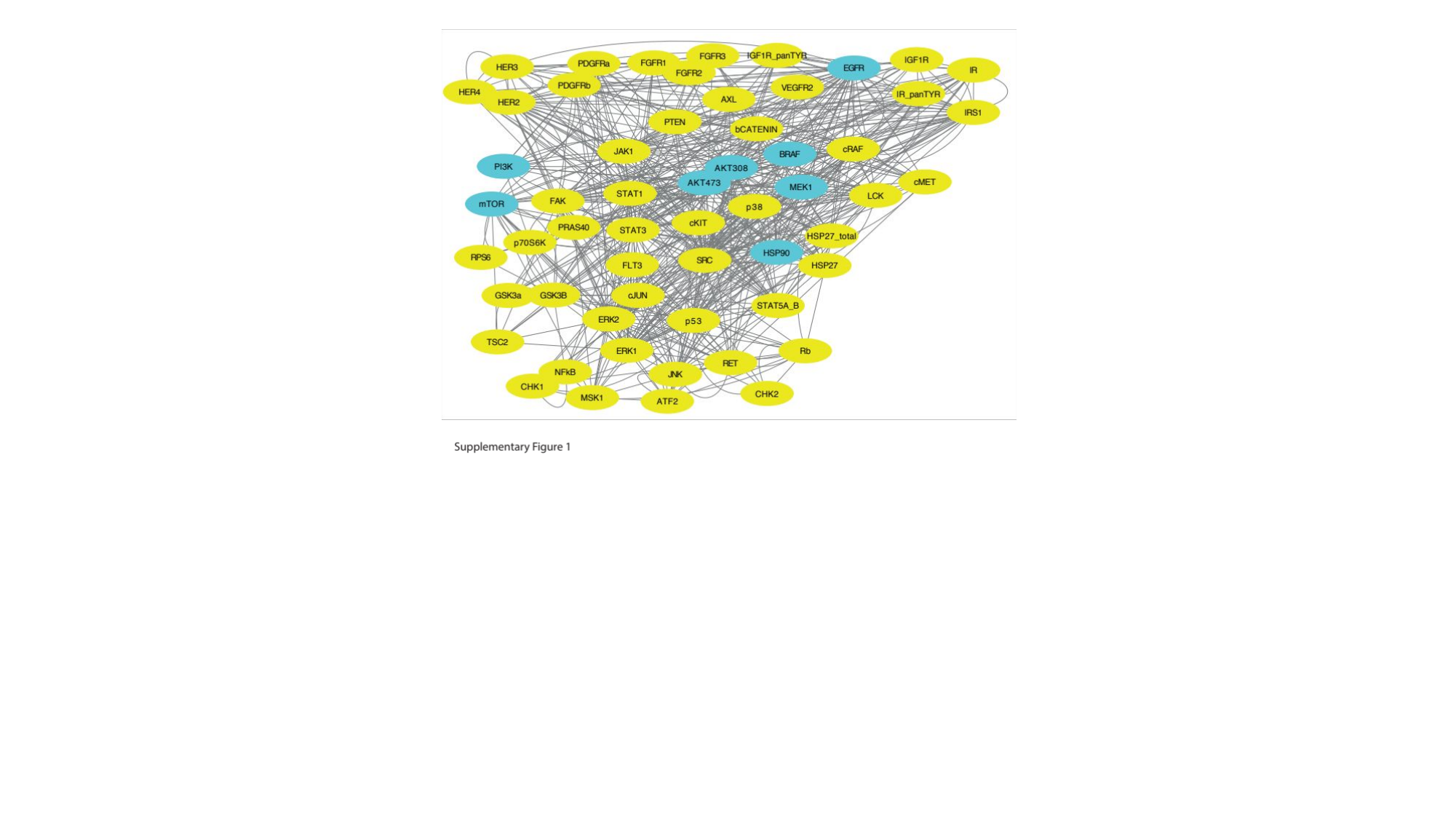

## Slide 2
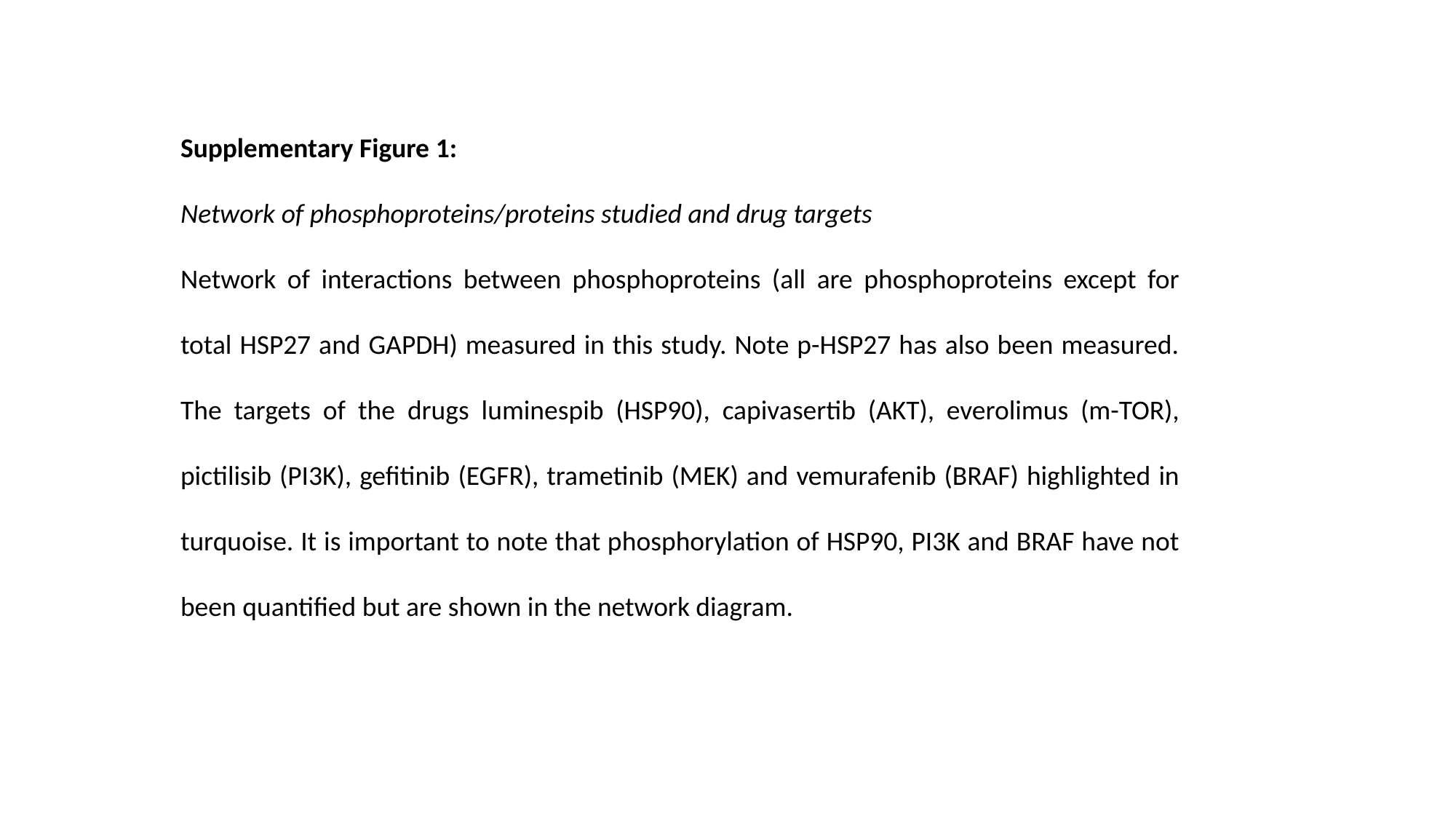

Supplementary Figure 1:
Network of phosphoproteins/proteins studied and drug targets
Network of interactions between phosphoproteins (all are phosphoproteins except for total HSP27 and GAPDH) measured in this study. Note p-HSP27 has also been measured. The targets of the drugs luminespib (HSP90), capivasertib (AKT), everolimus (m-TOR), pictilisib (PI3K), gefitinib (EGFR), trametinib (MEK) and vemurafenib (BRAF) highlighted in turquoise. It is important to note that phosphorylation of HSP90, PI3K and BRAF have not been quantified but are shown in the network diagram.
