# Supplementary material for: Individualized Prediction of Drug Response and Rational Combination Therapy in NSCLC Using Artificial Intelligence–Enabled Studies of Acute Phosphoproteomic Changes
Source: Mol Cancer Ther. 2022 Apr 3;21(6):1020–9. doi: 10.1158/1535-7163.MCT-21-0442 (PMC9381105; doi:10.1158/1535-7163.MCT-21-0442)
Supplement: Supplementary Figure [file mct-21-0442_supplementary_figure_2_suppsf2.pptx]

## Slide 1
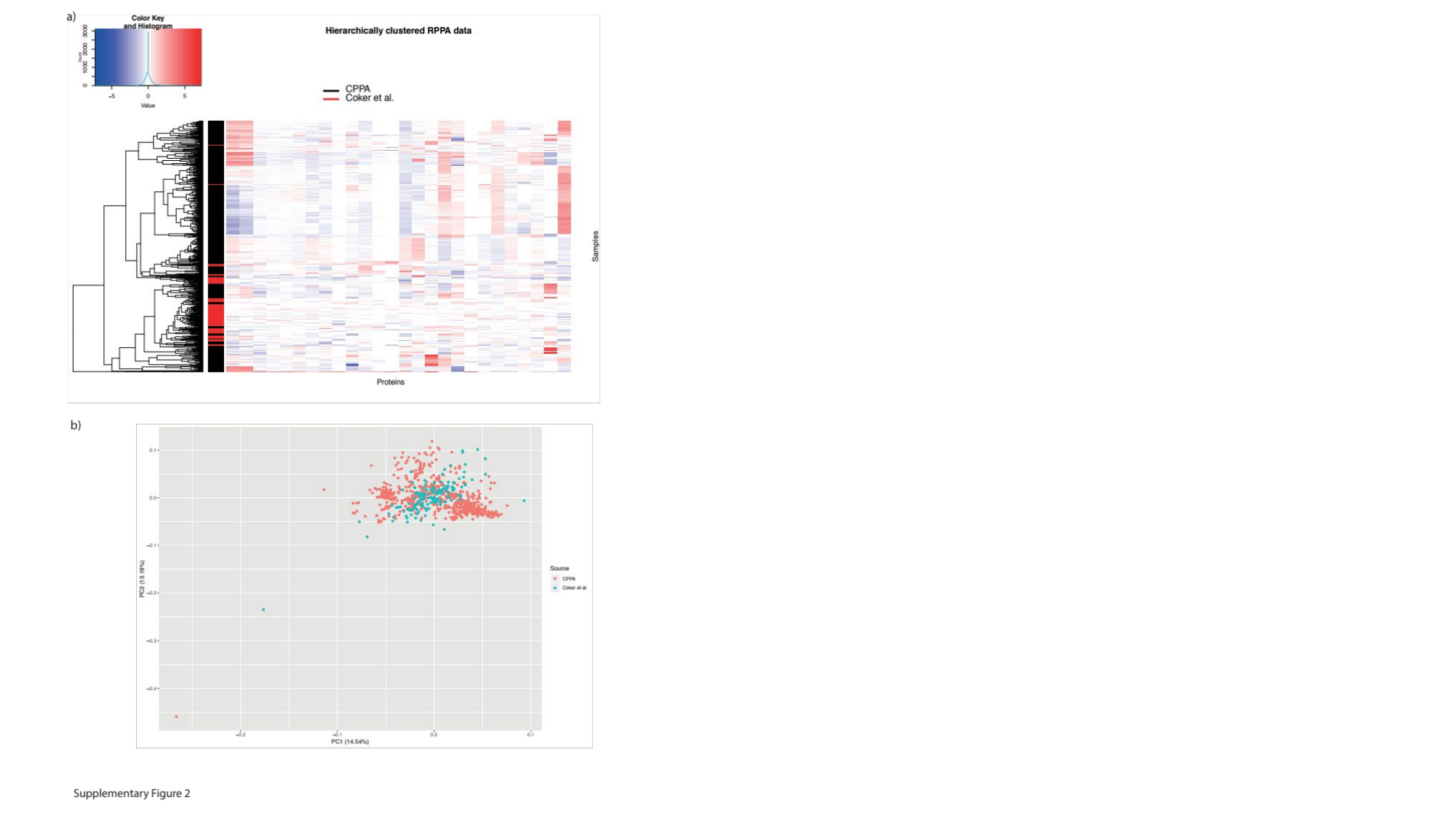

## Slide 2
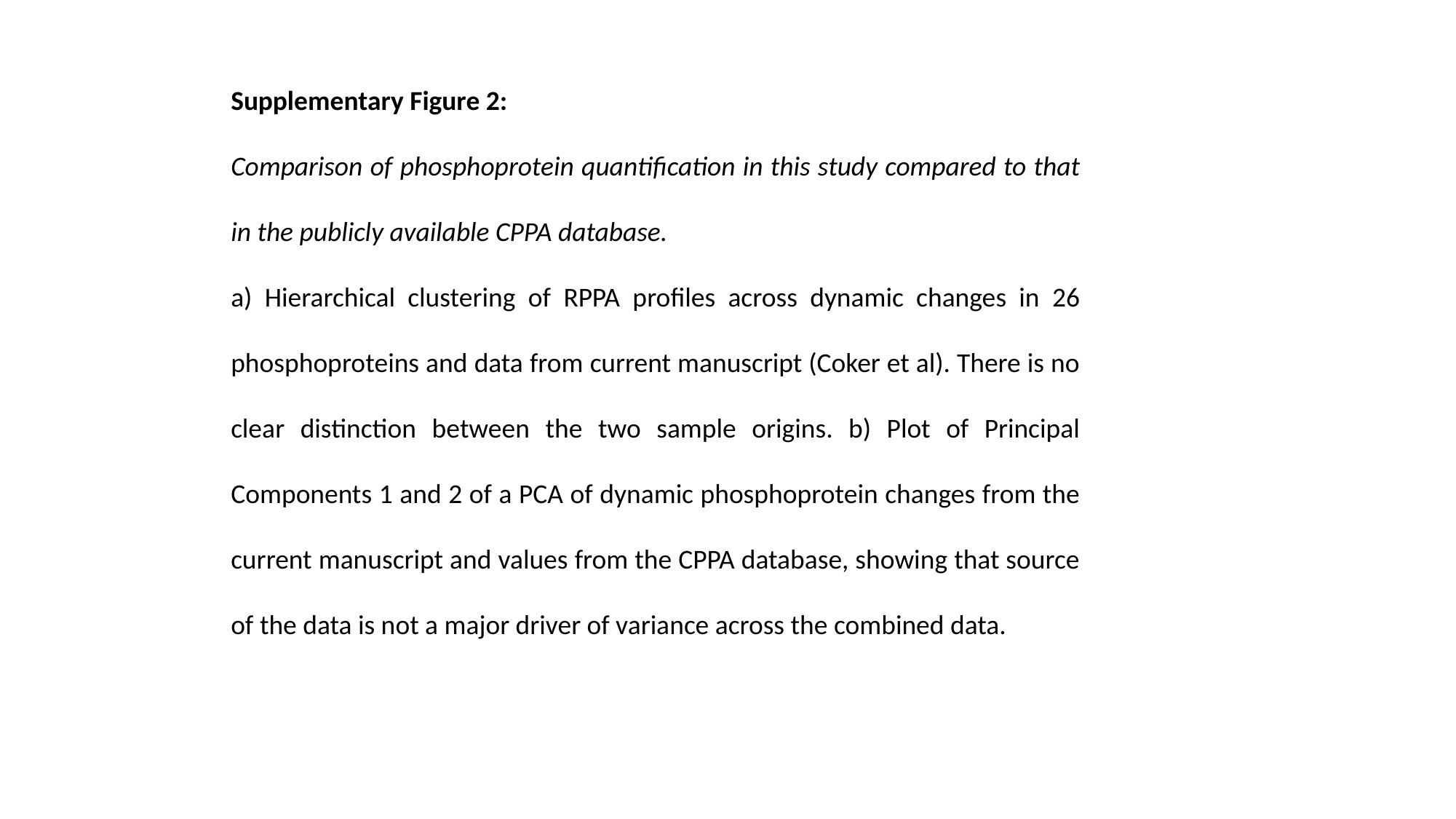

Supplementary Figure 2:
Comparison of phosphoprotein quantification in this study compared to that in the publicly available CPPA database.
a) Hierarchical clustering of RPPA profiles across dynamic changes in 26 phosphoproteins and data from current manuscript (Coker et al). There is no clear distinction between the two sample origins. b) Plot of Principal Components 1 and 2 of a PCA of dynamic phosphoprotein changes from the current manuscript and values from the CPPA database, showing that source of the data is not a major driver of variance across the combined data.
