# Supplementary material for: Individualized Prediction of Drug Response and Rational Combination Therapy in NSCLC Using Artificial Intelligence–Enabled Studies of Acute Phosphoproteomic Changes
Source: Mol Cancer Ther. 2022 Apr 3;21(6):1020–9. doi: 10.1158/1535-7163.MCT-21-0442 (PMC9381105; doi:10.1158/1535-7163.MCT-21-0442)
Supplement: Supplementary Figure [file mct-21-0442_supplementary_figure_3_suppsf3.pptx]

## Slide 1
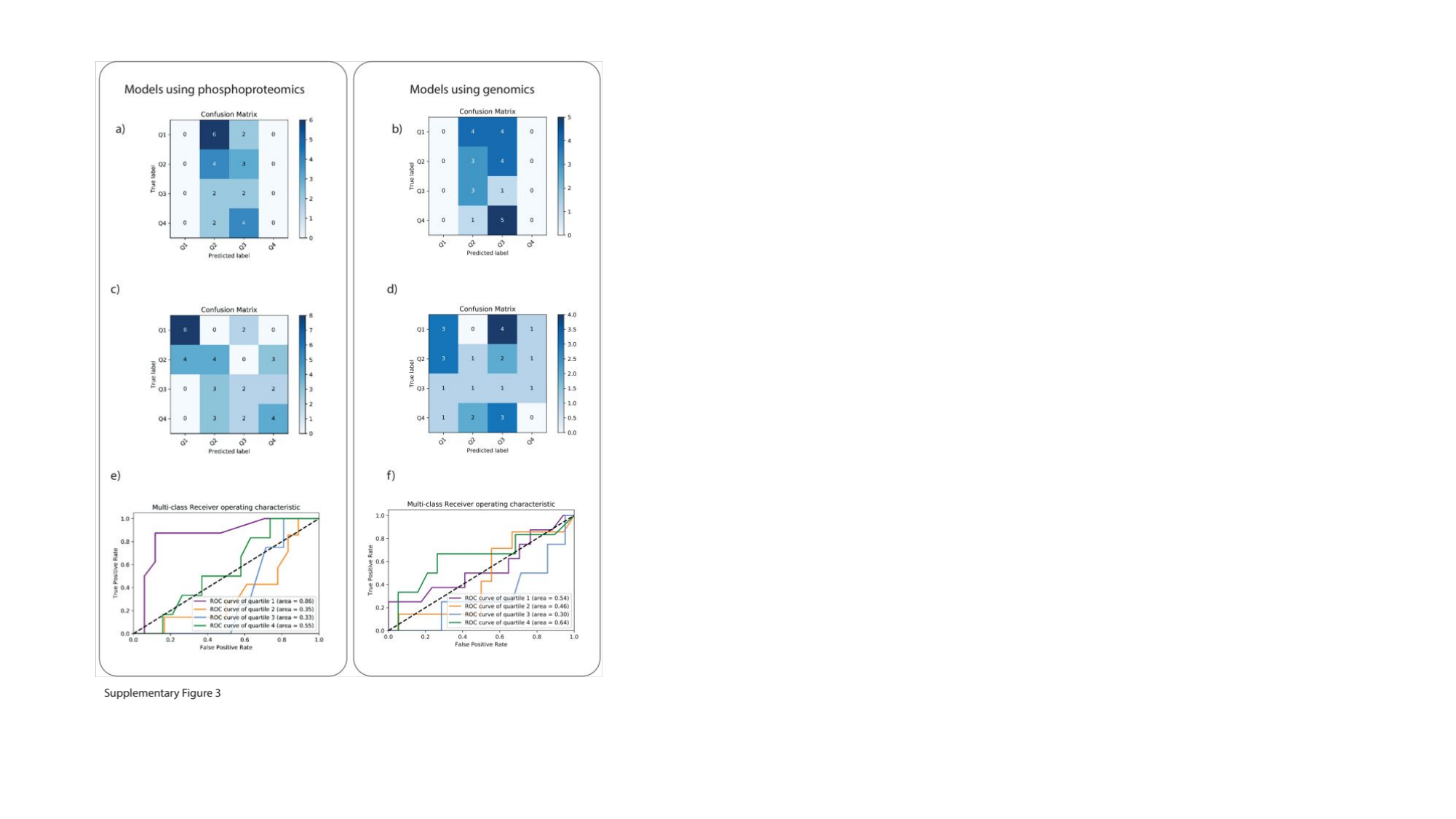

## Slide 2
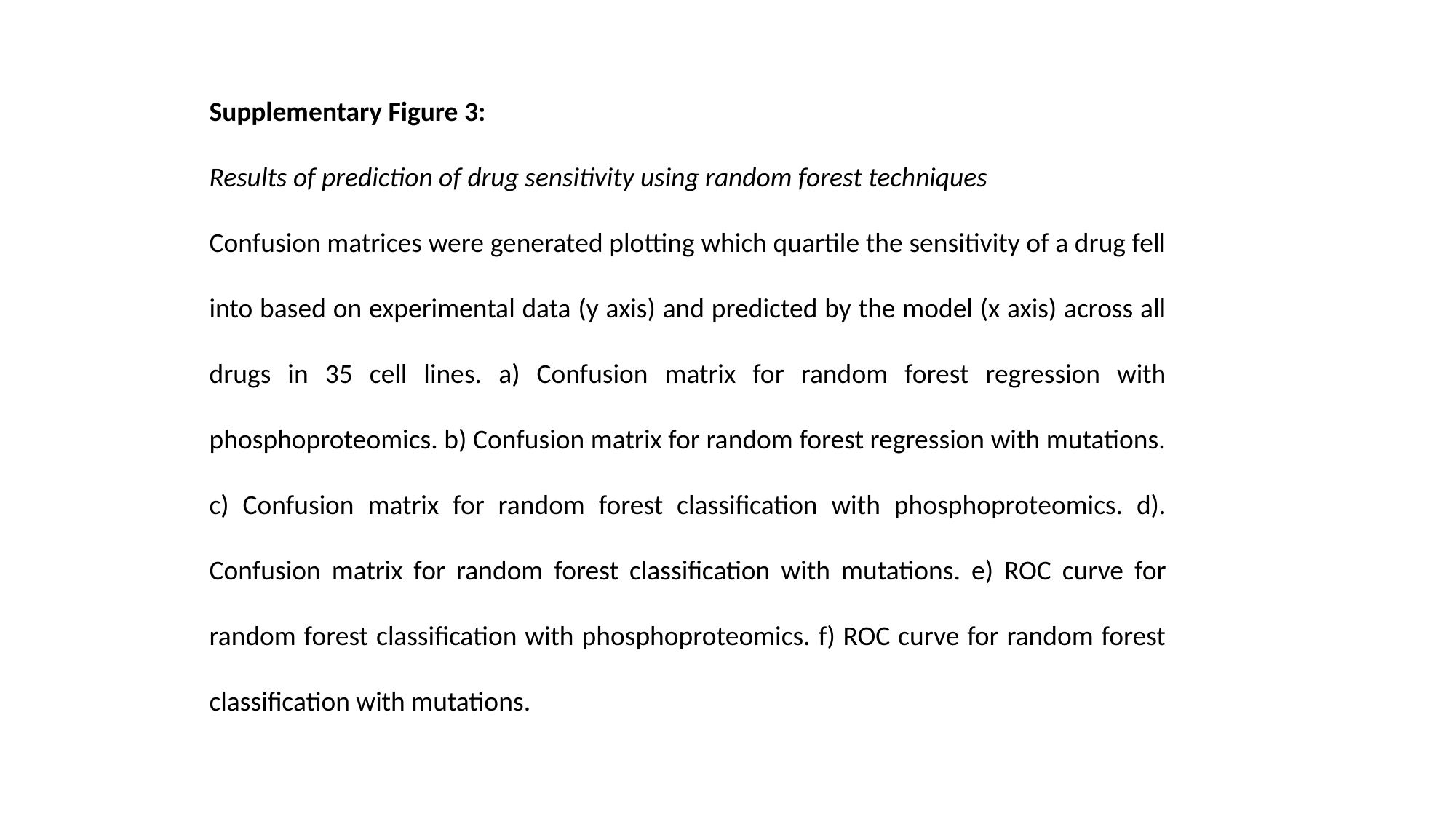

Supplementary Figure 3:
Results of prediction of drug sensitivity using random forest techniques
Confusion matrices were generated plotting which quartile the sensitivity of a drug fell into based on experimental data (y axis) and predicted by the model (x axis) across all drugs in 35 cell lines. a) Confusion matrix for random forest regression with phosphoproteomics. b) Confusion matrix for random forest regression with mutations. c) Confusion matrix for random forest classification with phosphoproteomics. d). Confusion matrix for random forest classification with mutations. e) ROC curve for random forest classification with phosphoproteomics. f) ROC curve for random forest classification with mutations.
