# Supplementary material for: Individualized Prediction of Drug Response and Rational Combination Therapy in NSCLC Using Artificial Intelligence–Enabled Studies of Acute Phosphoproteomic Changes
Source: Mol Cancer Ther. 2022 Apr 3;21(6):1020–9. doi: 10.1158/1535-7163.MCT-21-0442 (PMC9381105; doi:10.1158/1535-7163.MCT-21-0442)
Supplement: Supplementary Figure [file mct-21-0442_supplementary_figure_4_suppsf4.pptx]

## Slide 1
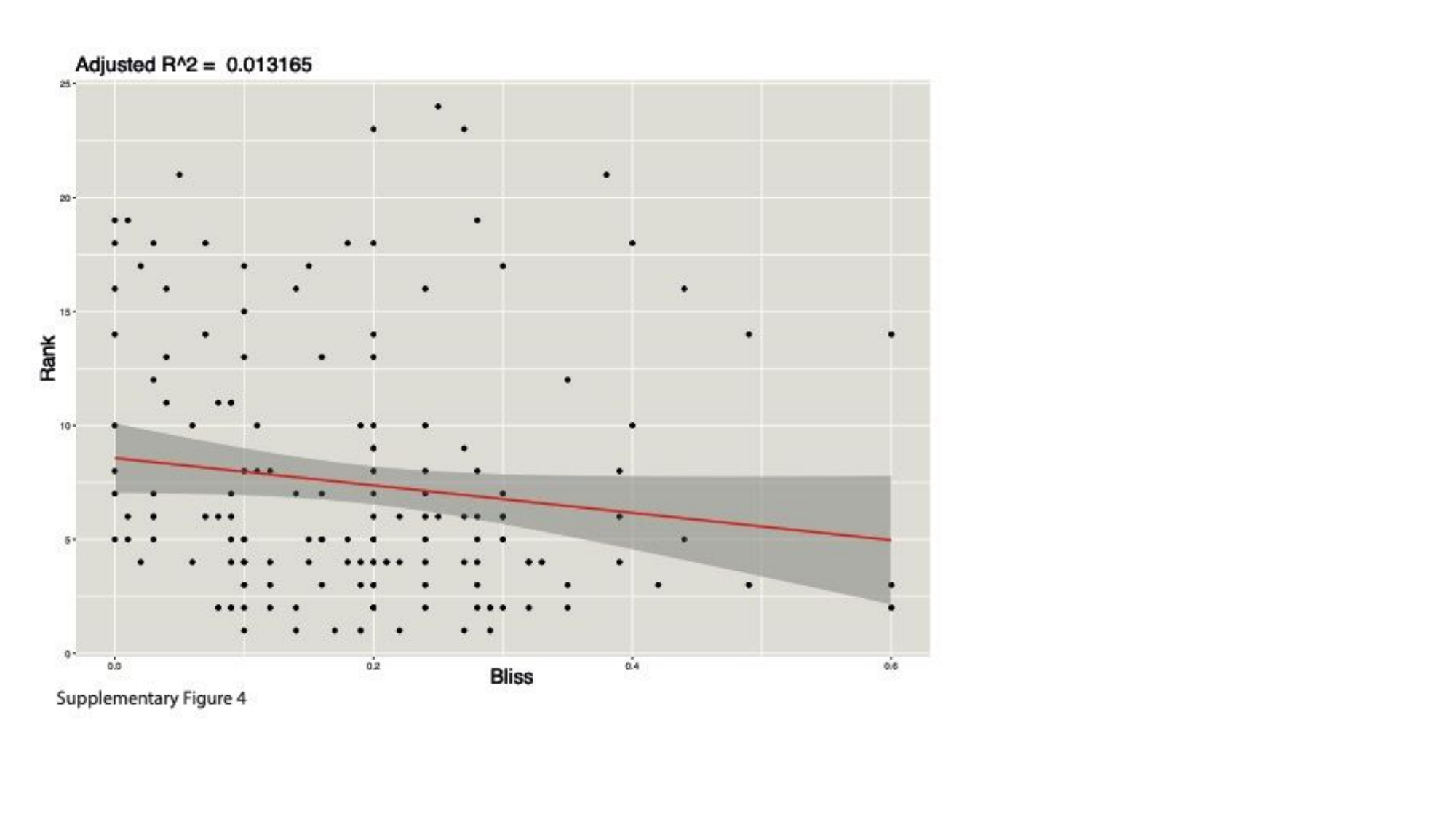

## Slide 2
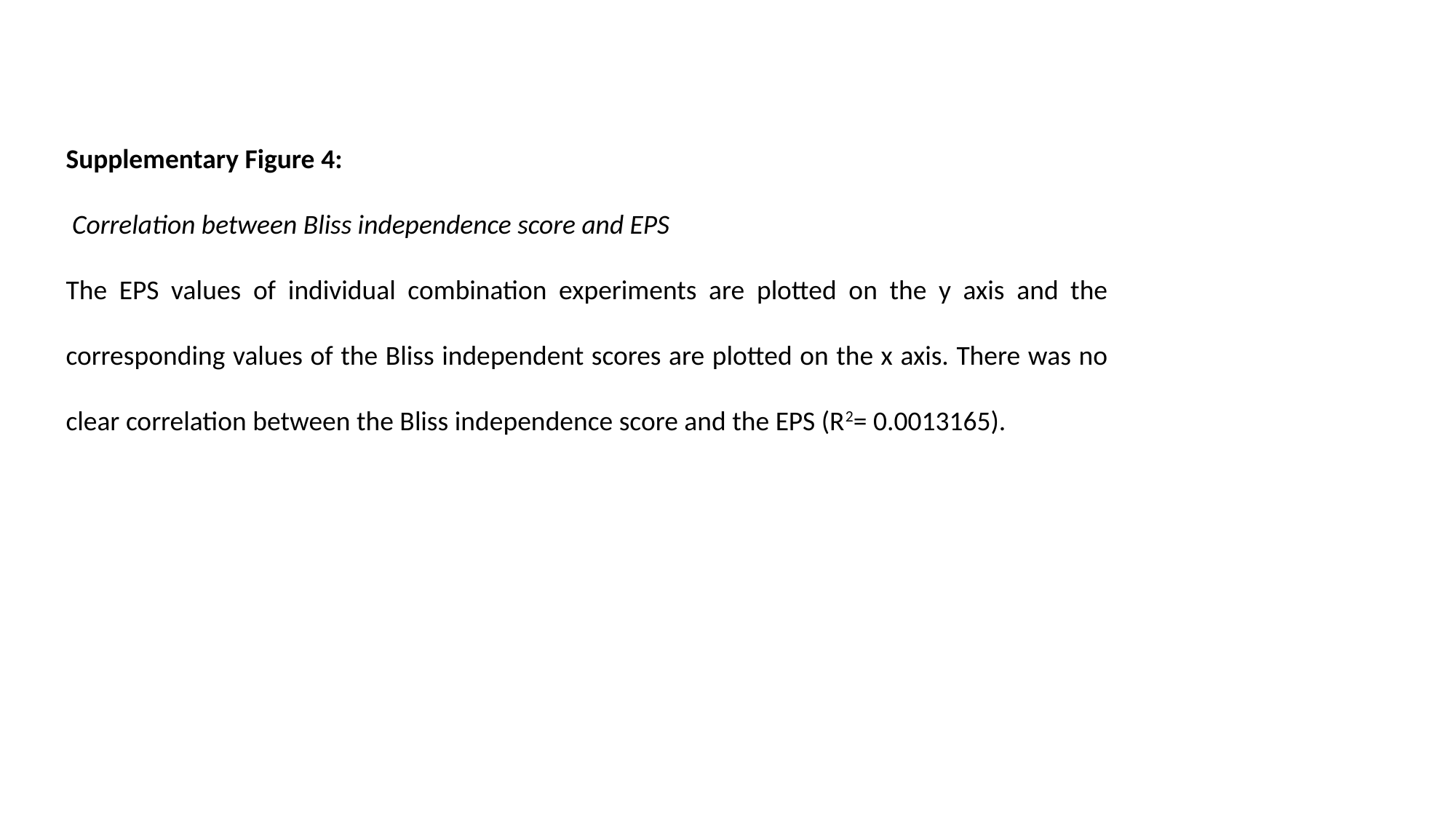

Supplementary Figure 4:
 Correlation between Bliss independence score and EPS
The EPS values of individual combination experiments are plotted on the y axis and the corresponding values of the Bliss independent scores are plotted on the x axis. There was no clear correlation between the Bliss independence score and the EPS (R2= 0.0013165).
