# Supplementary material for: Individualized Prediction of Drug Response and Rational Combination Therapy in NSCLC Using Artificial Intelligence–Enabled Studies of Acute Phosphoproteomic Changes
Source: Mol Cancer Ther. 2022 Apr 3;21(6):1020–9. doi: 10.1158/1535-7163.MCT-21-0442 (PMC9381105; doi:10.1158/1535-7163.MCT-21-0442)
Supplement: Supplementary Table [file mct-21-0442_supplementary_methods_and_table_legends_suppsmtl.docx]

**Supplementary Tables**

**Supplementary Table 1: Cell lines**

Cell lines and clinically relevant mutations

**Supplementary Table 2: Ability of *EGFR* mutations and *PIK3CA* mutations to predict response to Gefitinib and Pictilisib**

Contingency tables used for Chi-squared tests on sensitivity to (a) EGFR inhibitor gefitinib in *EGFR* mutant and wildtype cell lines. Chi-squared value with Yates correction = 0.1823, with 3 degrees of freedom, p = 0.669411. Non-significant p-value suggests being in quartile 1 or 2 (sensitive) are not more common in the *EGFR* mutated cell lines than expected purely due to chance. (b) PI3K inhibitor pictisilib in *PIK3CA* mutant and wildtype cell lines. Chi-squared value with Yates correction = 1.4644, with 3 degrees of freedom, p = 0.226238. Non-significant p-value suggests being in quartile 1 or 2 (sensitive) is not more common in the *PIK3CA* mutated cell lines than expected purely due to chance.

**Supplementary Table 3: Environmental perturbation scores**

The environmental perturbation scores of known drug targets (as reported by canSAR).

**Supplementary Table 4: Comparison of Bliss independence score and EPS of nodes**

The combination data are presented in descending order of Bliss independence scores. For each combination, EPS was calculated based on the phosphoprotein changes observed for the cell line treated with Drug 1. ‘Rank’ equates to the ranking of the EPS score for the target of the Drug 2, when the score of all nodes for the Drug 1 treatment are ranked from largest to smallest. For example, when EPS was calculated for HCCC827 with trametinib, the AKT308 node (a target of Capivasertib) was the node with the third highest EPS score in that network. Comparisons of Bliss score and EPS rankings are available for all phosphoprotein targets of Drug 2s.

# Supplementary Methods

Scripts and data are available at <https://github.com/eac54/SOCRATES>.

## Cell lines

A549, HCC2935, HCC827, NCI-H1373, NCI-H1437, NCI-H1563, NCI-H1568, NCI-H1693, NCI-H1734, NCI-H1781, NCI-H1838, NCI-H1944, NCI-H1975, NCI-H1993, NCI-H2030, NCI-H2228, NCI-H2291, NCI-H23, NCI-H2347, NCI-H441, NCI-H522, NCI-H820 and PC9 were all purchased from ATCC (LGC Standards, Teddington, UK). COR-L105, HCC4006, HCC44, NCI-H1650, NCI-H1755, NCI-H1793, NCI-H2087 and NCI-H3122 were all obtained from in-house sources. NCI-H1792, NCI-H2122, NCI-H358 and SK-LU-1 were kindly provided by The Francis Crick Institute Cell Services (London, UK).

All cell lines were grown in RPMI-1640 (11835-063, Gibco, Burlington, ON, Canada) except for SK-LU-1 which was grown in Dulbecco’s Modified Eagle’s Medium (D5671, Sigma-Aldrich). Additionally, all media was supplemented with 10% FBS (10270-106, Gibco), 1mM L-Glutamine (25030-024, Gibco) and 1x MEM non-essential amino acid solution (M7145, Sigma-Aldrich). Cells were incubated at 37 ^o^C with 5% CO_2_. All end point experiments were carried out in 20% FBS to more closely mimic the *in-vivo* environs.

## Isolation of cancer cells from NSCLC serous effusions

Up to 1000 ml of ascites or pleural fluid was collected by the patient and immunomagentically separated using previously published methods(1). In brief, samples were split into 250 mL flasks and centrifuged at 1,000 × g for 10 minutes at 4°C (Eppendorf 5810-R). The supernatant was removed and the pellet was resuspended in 1 mL then hybridized with 25 μL of EpCAM-coated Dynal beads (16102, Invitrogen), hybridized at 4°C for 30 minutes and magnetically separated according to the manufacturer’s instructions. Enriched cell pellets were seeded into 25cm^2^ TC flasks and dosed as per cell lines.

## Drugs and concentrations used

Drug concentrations used for our Luminex assays were based off the clinical maximum tolerable concentration (Cmax) normalised to the protein binding effect in 20% FBS media. Capivasertib (725nM), everolimus (0.52nM), gefitinib (28nM), luminespib (116nM), pictilisib (96nM), trametinib (1.81nM) and vemurafenib (317uM) were purchased from Selleck Chemicals LLC (S8019, S2247, S2807, S1120, S1025, S1069, S1065, S2673, S1267, Houston, TX, USA). All drugs were diluted in DMSO (D2680, Sigma-Aldrich) and stored for no longer than 6 months at -80^o^C. Patient-derived samples were not treated with vemurafenib or luminespib.

## Luminex magnetic bead suspension array

Cells were grown in 25cm^2^ tissue culture flasks (Corning Inc, New York, USA) at 20% FBS until approximately 80% confluent then dosed with one of seven drugs (plus 3 DMSO controls) for 1 hour. Lysate was stored at -80 ^o^C until required. MILLIPLEX MAP Akt/mTOR phosphoprotein kit, MILLIPLEX MAPK/SAPK signalling kit, MILLIPLEX MAP RTK phosphoprotein kit (48-611MAG, 48-660MAG, HPRTKMAG-01K respectively, MerckMillipore, Billerica, MA, USA) were combined with the following singleplex magnetic bead sets to produce three multiplex Luminex assays: phospho-NFkB, phospho-SRC, phospho-STAT3, phospho-STAT5 A/B, total HSP27 and GAPDH (46-702MAG, 46-710MAG, 46-623MAG, 46-641MAG, 46-608MAG, 46-667MAG, MerckMillipore). Bio-Plex Pro phospho-PDGFRa, phospho-PDGFRb and Akt (Thr308) (171-V50017M, 171-V50018M, 171-V50002, Bio-Rad, Watford, Herts, UK) were combined into a triplex assay. Manufacturer’s protocols were followed throughout.

Additionally, a home grown multiplex Luminex assay was created utilising a range of antibodies from Cell Signaling Technology (Danvers, MA, USA), targeting proteins of interest. These were conjugated to Luminex MagPlex Microspheres (MC100XX-01, Luminex, Austin TX, USA) via an xMAP Antibody Coupling Kit (40-50016, Luminex). A second set of antibodies, targeting phosphorylated versions of the antibodies from the first set were biotinylated (Biotin Type A conjugation kit, ab102865, Abcam, Cambridge, UK). This home-grown assay followed the Millipore protocol; however the unbound protein removal wash step was removed as it was found that this helped to reduce background signal from free biotin. Phosphoprotein levels were measured on the Luminex 200 system utilising xPONENT v3.1 software.

We attempted to quality control our Luminex platform; the coefficient of variance per analyte was calculated across three test cell lines (A2780, HT29 and NCI-H520). Each cell line was run in triplicate and each repetition was run across five separate 96 well plates. All plates were run in a single sitting to avoid inter-daily fluctuations. Baseline phospho-protein levels were measured and CVs calculated.

## Analysis of phopshoproteomic data

All phosphoproteomic data were normalized to GAPDH, which was then excluded from downstream analysis. This brought the total number of antibodies to 53, including: 2 antibodies for HSP27, as HSP27 & HSP27 (total); 2 antibodies for IGF1R, as IGF1R & IGF1R (pan); and 2 antibodies for IR, as IR & IR (pan). Importantly, for each cell line, three control samples and one sample for each drug treatment was set up. A standard deviation was calculated for each control and if the drug treated sample had a value more than 2 standard deviations above/below the control mean it was classified as increased/decreased. If it was within 2 standard deviations above or below the control it was considered unchanged. We chose to use dichotomous, increased, decreased or no change outputs as we had not validated the linearity of the absolute changes in phosphoprotein in our assays. We chose to run only controls and not treat samples in triplicate because of the cost. We have also calculated significance by looking at mean & standard deviation values, and created another file with only significant changes. However, by comparing "using all values & only significant values" we have decided on using "all values" for all of our analysis.

## Categorisation of data

Patient data is not categorised by sensitivity as it lacked sensitivity values (IC50s).

Cell line GI50 values for each drug were normalised to be between 0 and 1 based on the maximum and minimum value per drug. These values were then ranked and categorized values into four quartiles (Q1,Q2,Q3 and Q4): Q1 corresponds to the most sensitive quartile of cell lines for a given drug (lowest normalised sensitivity value), Q2 to the 25-50th percentiles, Q3 for 50th-75th percentiles, and Q4 for the least sensitive quartiles.

## Cytotoxicity studies

Growth inhibition was assessed via standard 72 hour Sulforhodamine B (SRB) assays. Briefly, cells were seeded into 96 well plates (353072, Corning Inc) and allowed to settle for 24 hours. A serial dilution for each drug was prepared and cells were dosed in quadruplicate at each concentration. After 72 hours plates were fixed in 10% trichloroacetic acid (10162590, Fisher Scientific, Waltham, MA, USA) for 20 minutes at 4 ^o^C before being stained with SRB dye (S9012, Sigma-Aldrich) for 20 minutes and left to dry. Re-solubilisation with 10mM Tris base solution (T1503, Sigma-Aldrich) allowed optical density determination at 570nm via an Infinite F50 plate reader (Tecan, Männedorf, Switzerland). GI50’s were calculated via GraphPad Prism v7.01 (La Jolla, CA, USA).

## 2D PCA

Principal component analysis was performed in R(2) using the vegan package(3).

## 3D PCA

Principal component analysis was performed in R using the pca3d package(4). For the plot of patient samples and cell lines, we have only used 5 drugs that were common in both patient and cell line datasets, i.e. luminespib and vemurafenib data were not used for this analysis.

## Heatmap of phosphoproteomic changes

This heatmap was created using Morpheus(5) using fold-changes of the cell lines. Two-way clustering of phosphoprotein changes and cell line+drug combinations is shown, based on Euclidean distances between protein change profiles. Values greater than or equal to 2 are coloured red, values less than or equal to 2 are coloured blue, and values between +2 and -2 are scales red-white-blue, with 0 corresponding to white.

## Unrooted Clustering of phosphochanges

The unrooted dendrogram was plotted using R, the ape R package(6) and Ward D2 clustering using Euclidean distances of ‘fold-changes’ proteomic data.

## Prediction & Classification

Recursive Feature Selection using Random Forest to predict Sensitivity values, using the R CARET package(7). This identified that 10 phosphoprotein values are good enough to make sensitivity predictions (100% true positive rate for Q1 and Q4).

We have variable importances as table (left table below); AKT phosphochanges were most important ones. (x is importance). We also have importances retrieved using Python (right plot). AKT still most important


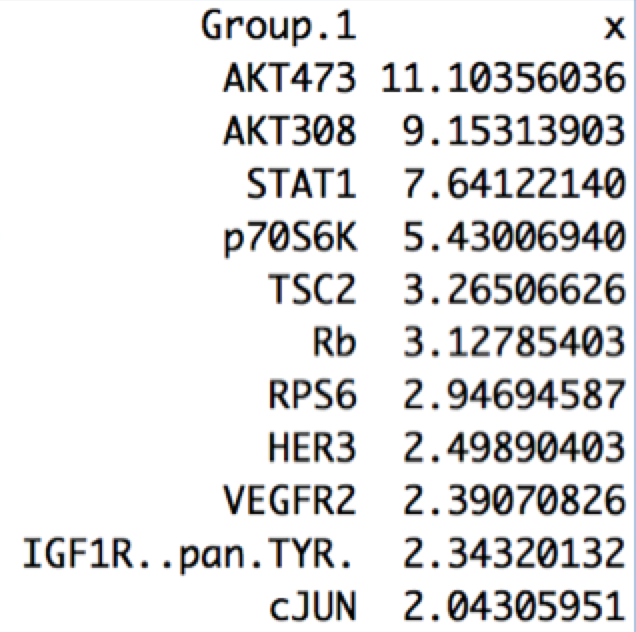


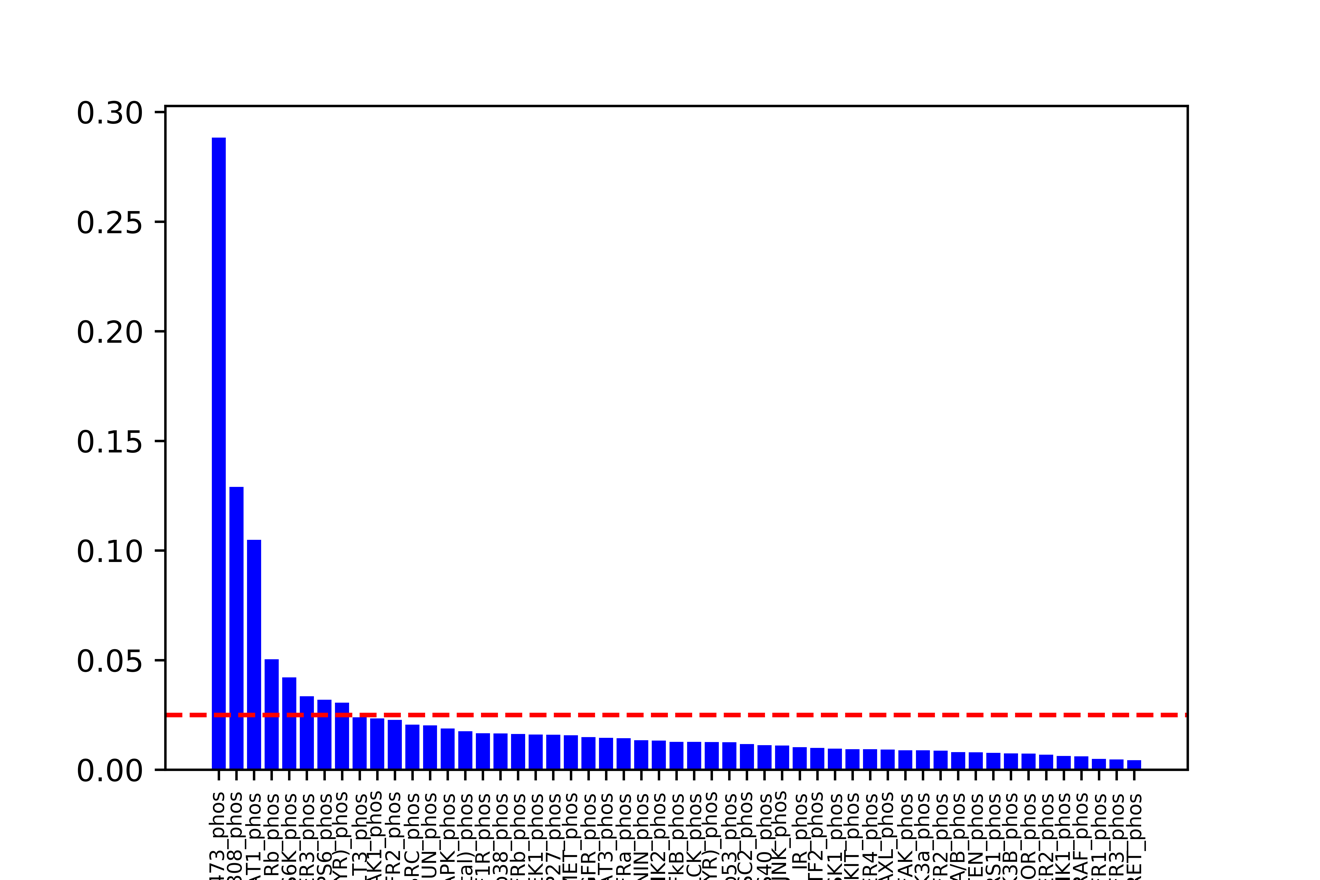


Done the above using the mutation data, and none of the samples were predicted to be in Q1 and Q4.

## Random Forest Regressor and Classifier

Using the 10 most important phosphoprotein values, we attempted to correctly classify cell lines into Q1 or Q4 based on their sensitivity to a given drug. This was achieved using Random Forest Regressor and Classifier in Python’s scikit-learn package(8).

With the random forest regressor we achieve classification with 100% specificity (True Positive Rate). To train the regressor we use all phosphovalues and quartile sensitivity values. To test, we used all phosphovalues to predict quartile sensitivity values. Predictions generated are a value between 1 and 4, and so we convert those continuous values to Q1, Q2, Q3 and Q4 categories. <1.5 is assigned to Q1. 1.5<=x<2.5 is assigned to Q2. 2.5<=x<3.5 is assigned to Q3. 3.5> is assigned to Q4. Confusion Matrix and R^2^ score of 0.87 is provided for classification assessment.

When we use Random Forest Classifier, we have to use distinct training/test datasets to avoid overfitting. We randomly selected 85% of the data as the training dataset and 15% as test dataset. As a result of classification on the test dataset, this produced an inferior specificity. For Q1 we still perform well when looked at ROC curves, however Q4 is not that good anymore. ROC curve and confusion matrix is provided for assessment of classification.

## Elastic Net Analysis

Elastic Net predictions of drug sensitivity were made using either phosphochanges or mutations. Mutations associated with each cell line were identified using the CCLE database (9). For mutational data, elastic net parameters (optimal alpha and l1_ratio) are decided using different values in different iterations. Optimal alpha was determined as: 0.11, l1_ratio as 0.1, and number of max iterations needed as 1. Using mutational data, classification performance was not better than random (everything predicted to be either Q2 or Q3 and the best area under the curve was 0.5). For phosphochanges, elastic net parameters (optimal alpha and l1_ratio) are decided using different values in different iterations. Optimal alpha is determined as 0.003, l1_ratio as 0.9, and number of max iterations needed as 24. Using phosphochanges, predictive results were better than mutations; however, they were still very poor compared to Random Forest results (best area under curve was for Q1, which was 0.79).

## Environmental perturbation scores

The local environment confluence score is a metric devised to describe the amount of proteomic change seen in the interactors of a given node, in addition to its own change. It requires two data types: firstly, a set of protein (node) values, here, the phopshoprotein abundance changes from the Luminex assay following treatment with a small molecule inhibitor. Secondly, the technique requires a list of edges between the nodes measured, forming a network connecting some or all of the nodes. The local perturbation score of a particular node (protein) is calculated as follows:


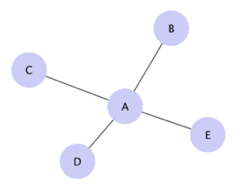


*Figure 1: example network*

$$Local environment score of A =\frac{\frac{Absolute value of A + \Sigma absolute value of A's interactors}{Degree of A}}{Range of values in entire network}$$

$$=\frac{\frac{\left| A \right| + \left| B\left| + \right|C\left| + \right|D \right| + \left| E \right|}{4}}{Range of values in entire network}$$

The degree of each node and the identity of its interactors are based on a network of binary interactions between proteins in the form of a protein-protein interaction network. By normalising to the range of values seen across the entire network, it is possible to compare local environment scores between different sets of proteomic measurements, i.e. different experimental conditions, drug treatments or between different patients Normalising to the degree also removes ‘study bias’: without this, a well-studied node with a large number of known interactors that show small changes in their absolute value in response to drug treatment may produce a higher local environment confluence score than a less well-characterised node with just one known interactor with a moderate or high absolute value change.

The interactome used for this was created using interactions between phosphoproteins in our panel as reported in the canSAR curated interactome(10). We had experiment results for 6 Cell Lines and 7 drugs. After calculating environmental perturbation scores for all nodes, only the scores of known drug targets (as reported by canSAR) were retained, as shown in Supplementary Table 3.

We have ignored results/predictions of luminespib, vemurafenib and pictisilib as targets of these drugs were not in our phosphopanel. We have also done an analysis including HSP90, BRAF and PI3KA in the interactome, however that only gave us worse results (ranking drop from 3.92 to 3.22).

After removing the luminespib, vemurafenib and pictisilib Bliss results, a histogram of Bliss values can be found below:


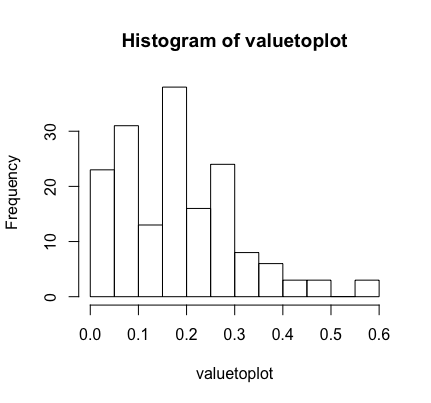


Performance is assessed by checking Q1 (25 samples w Bliss < 0.06) and Q4 Bliss (23 samples w Bliss >0.32) values (most synergistic and most non-synergistic) and how well we are making those predictions. Best performing method is Total, with ranking difference of “3.92” between Q1 and Q4. Which means we rank Q1 predictions ~4 ranking higher compared to Q4. For further significance, distribution of rankings for Q1 and Q4 are assessed initially using Mann-Whitney U Test. They were significantly different with p value of 0.003887. This p value of 0.003887 is then assessed further using the power of random permutations. 1000 permutations of randomly picking rankings for 23 samples, corresponding to number of samples in Q4 and 25 samples, corresponding to number of samples in Q1.


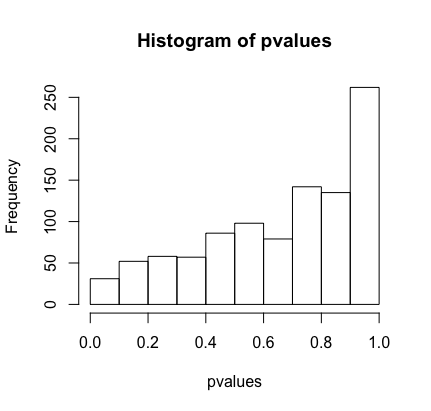


As a result, p-value of 0.01 is indeed very significant. Networks with mappings of environmental perturbation score were visualised using Cytoscape(11).

## Combination experiments

All cells were maintained in RPMI-1640 (11835-063) supplemented 10% FBS (Gibco 10270106), 2mM L-Glutamine (25030081), 1% NEAA (11140050) at 37°C and 5% CO2. For the combination experiments, cells were plated into a 384 well plate (Corning 3701). The outer wells of the plate were not used in order to reduce plate edge effects. The plating densities for each cell line had been predetermined in 384 well plates to allow linear logarithmic growth over the course of the experiment (96hrs). At plating, the percentage of FBS in the media was increased to 20% in order to mimic the C_max_ plasma concentration of drug *in vivo*. The cells were allowed to adhere overnight at 5%CO_2_/37°C. The two drugs were added sequentially using the Echo 525 acoustic dispenser. The first drug was added as a 10pt concentration response curve (DMSO 0.5% v/v) across the plate (see fig). The same volume of DMSO (0.5% v,v) was then backfilled onto the single agent concentration response curve and to the vehicle control and blank wells. The second drug was added by dispensing the compound in a concentration response curve vertically down the plate (see figure below).

**
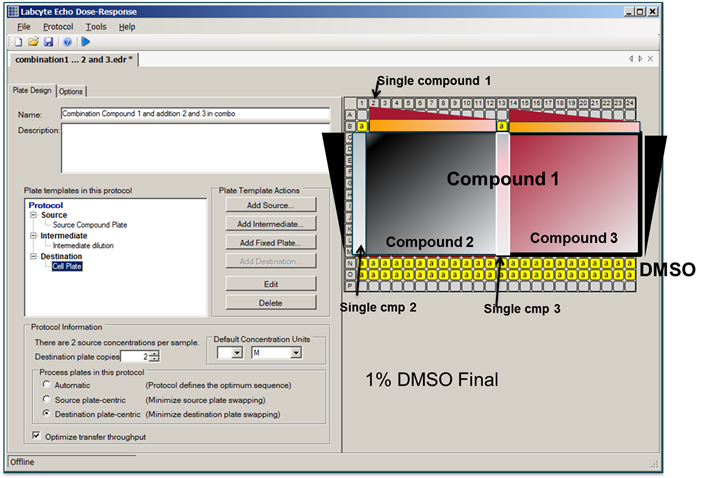
**

In this manner, 2 drug combinations could be conducted per 384 well plate. Again, the concentration of DMSO was maintained over the plate and the final concentration of DMSO was 1% (v,v) in all the wells. The plates were incubated for 72hrs at 5%CO_2_/37°C**.** The reagent CellTitre-Blue® (Promega) was used as the viability readout. 5µl was added and incubated for 3hrs at 37°C/5% CO_2_.The plate was read on EnVision plate reader (PerkinElmer). Data was plotted in heatmaps in R using package Superheat(12).

## Bliss Independence analysis

For analysis of the data, the Bliss Independence model(13) was chosen as the method of determining the synergistic effect of the combinations tested. The data was normalised to DMSO control. For each combination across the 6 different cell lines, the Z’ Factor was > 0.3 (screen mean 0.56) and the signal to background was >2.5 (mean 4.42). The following formula was used to calculate the Bliss score:

$$C=X-(\left( A+B \right)-\left( A*B \right))$$

Where

X = Fractional inhibition for combined drugs at given concentrations

A= Fractional inhibition for drug 1 at this concentration

B= Fractional inhibition for drug 2 at this concentration

A compound was classified as synergistic if it reached a Bliss score of >0.1.

**References**

1. Carden CP, Stewart A, Thavasu P, Kipps E, Pope L, Crespo M*, et al.* The association of PI3 kinase signaling and chemoresistance in advanced ovarian cancer. Mol Cancer Ther **2012**;11(7):1609-17 doi 10.1158/1535-7163.MCT-11-0996.

2. Team RC. R: A language and environment for statistical computing: R Foundation for Statistical Computing,Vienna, Austria; 2017.

3. Oksanen, J. et al. Vegan: community ecology package. R package version 1.17-4. URL http://CRAN. R-project. org/package= vegan (2010).

4. Weiner, J. pca3d: Three dimensional PCA plots. R package version 0. 8 484, (2015).

5. Morpheus. <https://software.broadinstitute.org/morpheus>.

6. Paradis E, Schliep K. ape 5.0: an environment for modern phylogenetics and evolutionary analyses in R. Bioinformatics **2019**;35(3):526-8 doi 10.1093/bioinformatics/bty633.

7. Kuhn Mea. Building predictive models in R using the caret package. J Stat Softw **2008**;28:1-26.

8. Pedregosa Fea. Scikit-learn: Machine Learning in Python. J Mach Learn Res **2011**;12:2825-30.

9. Ghandi M, Huang FW, Jane-Valbuena J, Kryukov GV, Lo CC, McDonald ER, 3rd*, et al.* Next-generation characterization of the Cancer Cell Line Encyclopedia. Nature **2019**;569(7757):503-8 doi 10.1038/s41586-019-1186-3.

10. Coker EA, Mitsopoulos C, Tym JE, Komianou A, Kannas C, Di Micco P*, et al.* canSAR: update to the cancer translational research and drug discovery knowledgebase. Nucleic Acids Res **2019**;47(D1):D917-D22 doi 10.1093/nar/gky1129.

11. Shannon P, Markiel A, Ozier O, Baliga NS, Wang JT, Ramage D*, et al.* Cytoscape: a software environment for integrated models of biomolecular interaction networks. Genome Res **2003**;13(11):2498-504 doi 10.1101/gr.1239303.

12. Barter RL, Yu B. Superheat: An R package for creating beautiful and extendable heatmaps for visualizing complex data. J Comput Graph Stat **2018**;27(4):910-22 doi 10.1080/10618600.2018.1473780.

13. CI B. The toxicity of poisons applied jointly. Ann Appl Biol **1939**;26:585–615.
