# Supplementary figures and images for: Individualized Prediction of Drug Response and Rational Combination Therapy in NSCLC Using Artificial Intelligence–Enabled Studies of Acute Phosphoproteomic Changes
Source: Mol Cancer Ther. 2022 Apr 3;21(6):1020–9. doi: 10.1158/1535-7163.MCT-21-0442 (PMC9381105; doi:10.1158/1535-7163.MCT-21-0442)

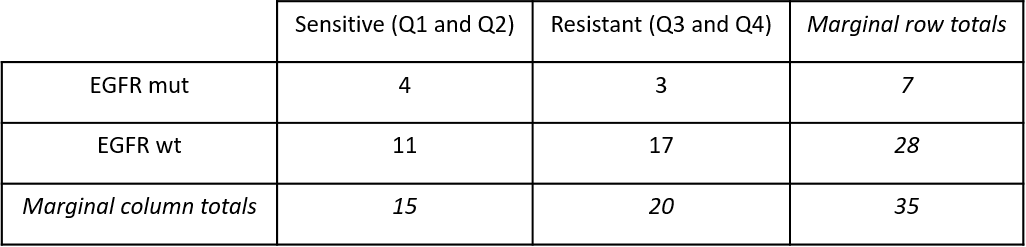

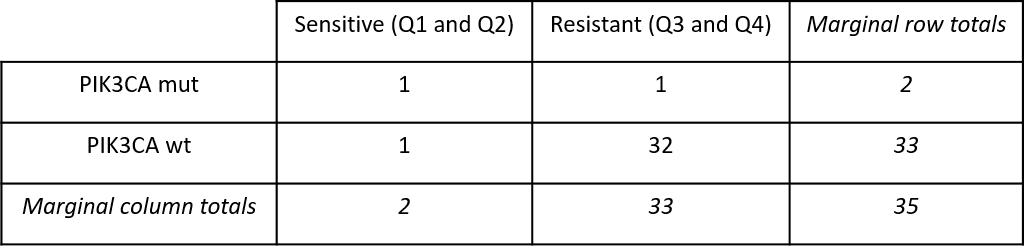


b)

a)

**Supplementary Table 2**

Supplement: Supplementary Table [file mct-21-0442_supplementary_table_2_suppst2.docx]
